# LECT 2 Antagonizes FOXM1 Signaling via Inhibiting MET to Retard PDAC Progression

**DOI:** 10.3389/fcell.2021.661122

**Published:** 2021-04-15

**Authors:** Xin Li, Pingping Lin, Ye Tao, Xin Jiang, Ting Li, Yunshan Wang, Chenjing Wang, Yu Cao

**Affiliations:** ^1^Department of Pharmacy, The Affiliated Hospital of Qingdao University, Qingdao, China; ^2^Department of Clinical Laboratory, The Second Hospital of Shandong University, Jinan, China

**Keywords:** PDAC, LECT2, HGF/MET, FOXM1 signaling, tumor growth

## Abstract

Pancreatic ductal adenocarcinoma (PDAC) is one of the most lethal cancers with minimally effective treatments, highlighting the importance of developing novel biomarkers and therapeutic targets. Here, we disclosed the mechanisms that leukocyte cell-derived chemotaxin-2 (LECT2) modulates PDAC development using in vitro and in vivo models. LECT2 is downregulated in metastatic PDACs compared with the primary tumor, and its expression is correlated with multiple clinical pathologic features and prognosis. The absence promotes multiple malignant behaviors, including cell proliferation, epithelial-mesenchymal transition, migration, and invasion. In vivo studies showed that LECT2 overexpression inhibits tumor growth and lung metastasis. Mechanistically, LECT2 inhibits FOXM1 signaling by targeting HGF/MET to retard PDAC progression, revealing LECT2 as a promising biomarker and therapeutic target for PDAC in the future.

## Introduction

Pancreatic ductal adenocarcinoma (PDAC) is the most common form of pancreatic cancer worldwide. Despite the progress in surgical techniques and medical treatment, the overall prognosis is still weak, as the 5-year relative survival rate is only 9% (Siegel et al., 2020). It is typically diagnosed at a distant stage resulting in limited treatment options, and the available therapies are mostly unsuccessful due to frequent recurrence and metastasis. In this case, there is an urgent need to identify new biomarkers or therapeutic targets to improve the diagnosis and prognosis of this malignancy.

As a 16-kDa secreted protein, leukocyte cell-derived chemotaxin-2 (LECT2) was initially isolated in humans from phytohemagglutinin-stimulated T-cell leukemia SKW-3 cells and identified as a chemotaxin of neutrophils (Yamagoe et al., 1996; Ito et al., 2003). Recently, it has been reported to have a batch of other functions in many pathological conditions, including arthritis (Okumura et al., 2008), cancer (Chen et al., 2014; Okabe et al., 2014), immune modulation (Ando et al., 2012; Lu et al., 2013), liver fibrosis (Xu et al., 2019), neuronal development (Koshimizu and Ohtomi, 2010), glucose metabolism and metabolic syndrome (Lan et al., 2014; Hwang et al., 2015). In terms of cancer, LECT2 has been shown to act as a tumor suppressor in multiple cancers, such as hepatocellular carcinoma, non-small cell lung cancer, and intestinal cancer (Chen et al., 2014, 2016; Greenow et al., 2018; Hung et al., 2018; L’Hermitte et al., 2019). Moreover, in combination with CYP1A1 and CETN1, LECT2 could be predictive for breast carcinoma recurrence, and it can also be predictive of survival among female smokers (Andres et al., 2015). However, the role of LECT2 in the development of PDAC and the underlying mechanisms are not entirely understood.

In this study, LECT2’s expression was detected in tissue samples from *in situ* and metastatic PDACs, which is related to the prognosis and clinical pathological characteristics. We also assessed the suppressive effect of LECT2 on various malignant behaviors of PDAC cell lines, both in vivo and in vitro. Besides, potential signaling pathways and target proteins involved in the molecular mechanism underlying the regulatory effect of LECT2 were also investigated. This work may give some clues to the potential of LECT2 as a biomarker or therapeutic target in tumor formation and metastasis of PDAC.

## Materials and Methods

### Cell Lines

Human pancreatic cancer cell lines HPNE, S2-007, BxPC3, S2-013, Panc-1, MiaPaca-2, and AsPC-1 were purchased from the ATCC (Amerian Type Culture Collection, ATCC). HPNE, BxPC3, Panc-1, S2-007, and S2-013 cells were cultured in DMEM (Biological Industries, Cat. No. 06-1055-57-1A) + 10% FBS (Biological Industries, Cat. No. 04-007-1A) + 1% penicillin/streptomycin (Solarbio, P1400); MiaPaca-2 and AsPC-1 cells were cultured in RPMI-1640 (Biological Industries, Cat. No. 01-101-1A) + 10% FBS (Biological Industries, Cat. No. 04-007-1A) + 1% penicillin/streptomycin. All cells were cultured at 37°C with 5% CO2 and saturated humidity.

### Antibodies and Reagent

Leukocyte cell-derived chemotaxin-2 antibody (Cat. No. ab119429), FOXM1 antibody (Cat. No. ab17379), MET antibody (Cat. No. ab51067), Cyclin D1 antibody (Cat. No. ab134175), Cyclin B1 antibody (Cat. No. ab32053), c-Myc antibody (Cat. No. ab185656), and Fibronection antibody (Cat. No. ab32419) were purchased from Abcam. E-cadherin antibody (Cat. No. #14472), β-catenin antibody (Cat. No. #8480), N-cadherin antibody (Cat. No. #13116), N-cadherin antibody (Cat. No. #13116), Vimentin antibody (Cat. No. #5741), Phospho-Met (Tyr1234/1235) antibody (Cat. No. #3077), mouse IgG antibody (Cat. No. #7076), and rabbit IgG antibody (Cat. No. #7074) were purchased from CST (Cell Signaling Technology). Hepatocyte Growth Factor (HGF) (Cat. No. H0536) was purchased from Merck. Selumetinib (AZD6244) (S1008) was purchased from Selleck.

### Plasmids and Short Hairpin RNAs

The cDNAs of LECT2 and FOXM1 were obtained from GeneCopoeia, and the cDNAs were constructed on a lentiviral vector. The shRNA sequence of LECT2 and FOXM1 was obtained from the Sigma website: shLECT2 #1: CCGGGCAGAAA GTTTATCCTGGCATCTCGAGATGCCAGGATAAACTTTCT GCTTTTTTG; shLECT2 #2: CCGGTTCTACATTAAGCCAA TTAAGCTCGAGCTTAATTGGCTTAATGTAGAATTTTTTG; shFOXM1: CCGGTTGCAGGGTGGTCCGTGTAAACTCGAGT TTACACGGACCACCCTGCAATTTTTG. The shRNA sequence was synthesized and constructed on a lentiviral vector.

### Western Blotting

The cells were lysed by RIPA lysate (Beyotime Biotechnology, Cat. No. P0013B) containing protease inhibitor (Roche, Cat. No. 11206893001), and the protein supernatant was collected after centrifugation; the protein supernatant was added to the protein loading buffer to denature the protein. Prepare 10 or 8% SDS-PAGE gel. After the protein is electrophoresed, transferred, and blocked, add the corresponding primary antibody and incubate at 4°C overnight. After washing, add the corresponding secondary antibody (CST, #7076, or #7074) and incubate at room temperature for 1 h. After washing, add ECL (Millipore, Cat. No. WBULS0500) for exposure; use the BioImaging Systems instrument to obtain protein expression pictures, and use ImageJ software to count gray values. Ratio to β-Actin % represents the ratio of the gray value of LECT2 to the gray value of β-Actin and then multiplied by 100.

### qRT-PCR

The cells were extracted with total RNA by the Trizon (ThermoFisher, A33250) method and reverse transcribed into cDNA using EasyScript^®^ Reverse Transcriptase (TRAN, AE101-02). Perform qRT-PCR with the following primers: 5′-CCAATGAGATCCGGACGTGT-3′ (LECT2 Forward) and 5′-TCCTGGCCCACAATCATTCC-3′ (LECT2 Reverse); 5′-GA GAAGGCTGGGGCTCATTT-3′ (GAPDH Forward) and 5′-A GTGATGGCATGGACTGTGG-3′ (GAPDH Reverse); 5′-GGGG TCTGTCATGGAAGGTG-3′ (E-cadherin Forward) and 5′-CA AAATCCAAGCCCGTGGTG-3′ (E-cadherin Reverse); 5′-AGG CGTTATGTGTGTATCTTCACT-3′ (N-Cadherin Forward) and 5′-GGAGGGATGACCCAGTCTCT-3′ (N-Cadherin Reverse); 5′-TCGTGCTTTGACCCCTACAC-3′ (Fibronection Forward) and 5′-CGGGAATCTTCTCTGTCAGCC-3′ (Fibronection Reverse); 5′-GGACCAGCTAACCAACGACA-3′ (Vimentin Forward) and 5′-AAGGTCAAGACGTGCCAGAG-3′ (Vimentin Reverse). The ratio to GAPDH represents the number of cycles of LECT2 minus the number of cycles of GAPDH.

### Immunohistochemistry

Sections of pancreatic cancer tissues were dewaxed, citric acid antigen repaired, and 3% hydrogen peroxide treated. Tissues were removed and then serum-blocked and antibody-incubated. Tissue-stained sections were analyzed by Image-Pro software for positive or negative areas.

### MTT and Cell Proliferation

Cells were counted and seeded in 96-well plates. After 48 h of culture, MTT (0.5 mg/ml) (Solarbio, M8180) was added and incubated for 4 h. 150 μl DMSO was added and mixed to obtain the OD value. After counting, the cells were plated in 6-well plates, cultured for 14 days. Fresh medium was changed twice a week. Cells were fixed with methanol, and crystal violet stained. The number of cell clones was photographed to count.

### Migration and Invasion

Transwell was covered with Migration gel and incubated at 37°C for 30 min. After adding an appropriate number of cells for 12 h, the cells were fixed and stained, and then the number of cell invasions was counted. After counting the cells, they were seeded in the Transwell cell in a 24-well plate, fixed for staining after 12 h in culture, and then counted the number of cell migration. Relative migration or invasion cells/per area means selecting five areas randomly and counting the number of cells in them.

### *In vivo* Tumorigenesis and Metastasis Assay

Collect pancreatic cancer cell lines that overexpress or knock LECT2, count and inject them into the skin of nude mice, and measure the xenograft tumor volume every week; The pancreatic cancer cell lines that overexpressed or knocked LECT2 were collected and injected into the tail vein of nude mice. Thirty days later, the lung tissues of nude mice were collected to observe the pancreatic cancer cell lung metastasis.

### RNA-Seq Analysis

For the BXPC3 cell line overexpressing LECT2, total RNA was extracted by Triton. Total RNA was sequenced using Illumina HiSeq and subsequently matched to the genome using TopHat2 software. Differential genes were analyzed using HTseq software and DESeq2 software. The RNA sequencing data has been uploaded to the GEO database (GSE168611).

### Specimens

Tissue samples from patients with pancreatic cancer were collected by the General Surgery of the Affiliated Hospital of Qingdao University, and pancreatic cancer *in situ* and metastases were recorded. Metastases are PDAC with lymph node metastasis. Pancreatic cancer tissue is stored in liquid nitrogen. The staging of pancreatic cancer patients strictly follows the American Cancer Society, the American Joint Committee on Cancer TNM system staging standards. Twenty-two primary (*in situ*) and twenty-two metastatic (Met) PDACs tissue samples were used to extract total RNA and total protein, and detect the mRNA and protein expression levels of LECT2. Besides, the expression of LECT2 in PDACs was also detected by immunohistochemistry. The cancer tissue used complies with the regulations of the Ethics Committee of Qingdao University.

### Statistical Analysis

All data were statistically analyzed using the Student’s *t*-test in SPSS software. Data are presented as mean ± SD, and *p* < 0.05 was considered statistically significant.

## Results

### LECT2 Expression Is Downregulated in Metastatic PDACs Compared With Primary Tumor

**FIGURE 1:**
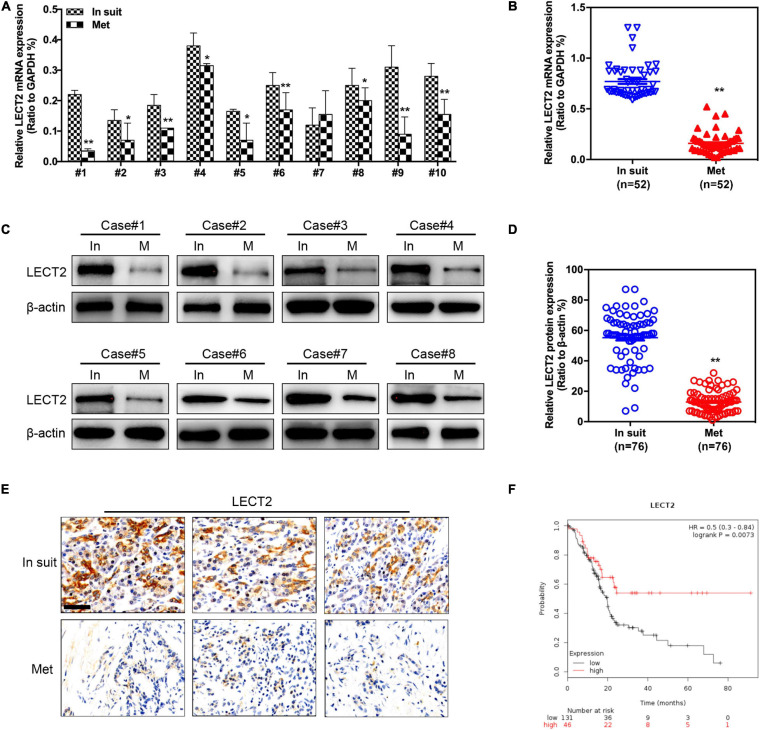
LECT2 expression in primary (*in situ*) and metastatic (Met) PDACs. **(A)** Representative qRT-PCR results of LECT2 expression in paired primary and metastatic (Lymph node metastasis) PDACs. **(B)** Quantification of qRT-PCR results in paired primary (*n* = 52) and metastatic (*n* = 52) PDACs. **(C)** Representative immunoblotting results of LECT2 protein levels in paired primary and metastases (Lymph node metastasis) PDACs. **(D)** Quantification of immunoblotting results in paired primary (*n* = 76) and metastatic (*n* = 76) PDACs. **(E)** Representative images of immunohistochemistry (IHC) staining for LECT2 in sections from paired primary and metastatic PDACs (Scale bar: 100 μm). **(F)** Kaplan-Meier analysis indicating the overall survival of PDAC patients with low (black) or high (red) LECT2 expression. *p < 0.01 and **p < 0.01.

The LECT2 expression was measured in paired primary tumors and metastatic tumors (Lymph node metastasis), respectively (Figures 1A,B). *In situ*, primary tumors showed higher mRNA levels of LECT2 than paired metastatic samples. Similarly, we also found that LECT2 protein levels were remarkably higher in primary PDACs compared with those of the paired metastatic specimens (Figures 1C–E). Moreover, Kaplan-Meier analysis indicated that low levels of LECT2 predicted poor clinical outcome in PDAC (Figure 1F). LECT2 expression was also correlated with multiple clinical pathologic features, including TNM staging, tumor size, lymph node metastasis, and distant metastasis (Table 1 and Supplementary Figure 1A). Moreover, the expression level of LECT2 is negatively correlated with tumor size, lymph node metastasis, and distant metastasis. Collectively, the results mentioned above implied that LECT2 might play a tumor-suppressive role in PDAC progression. Finally, we also analyzed the expression level of LECT2 in the PADC tumor and the adjacent area of the tumor. Immunohistochemical staining showed that the expression level of LECT2 in the tumor was significantly lower than that in the adjacent area of the tumor (Supplementary Figure 1B).

**TABLE 1:** Correlations between LECT2 expression and clinicopathologic features in PDCA patients.

Variable	Expression of LECT2		Total	*P*^*a*^
	Low	High		
**Age (years)**				
°≤65	65	31	96	0.163
°>65	110	37	147	
**Gender**				
°Male	32	98	130	0.317
°Female	25	68	113	
**Tumor location**				
°Head	115	44	159	0.102
°Body tail	63	19	84	
**TNM (AJCC)**				
°I, II	104	61	165	0.026
°III, IV	53	25	78	
**Tumor size**				
°≤3 cm	39	42	81	<0.001
°>3 cm	113	49	162	
**Tclassification**				
°T1, 2	98	44	142	0.076
°T3, 4	77	24	101	
**Lymph node metastasis**				
°Absent	95	81	176	0.012
°Present	54	13	67	
**Distant metastasis**				
°Absent	114	103	217	<0.001
°Present	22	4	26	
**Vascular Invasion**				
°Absence	132	77	209	0.253
°Present	26	8	34	
**Histological differentiation**				
°Well/Moderate	121	40	161	0.321
°Poor	61	22	83	

*^*a*^*p*-value < 0.05 was considered statistically significant. *p*-values were calculated using the Pearson chi-square test.*

### LECT2 Inhibits PDAC Tumor Growth Both *in vitro* and *in vivo*

**FIGURE 2:**
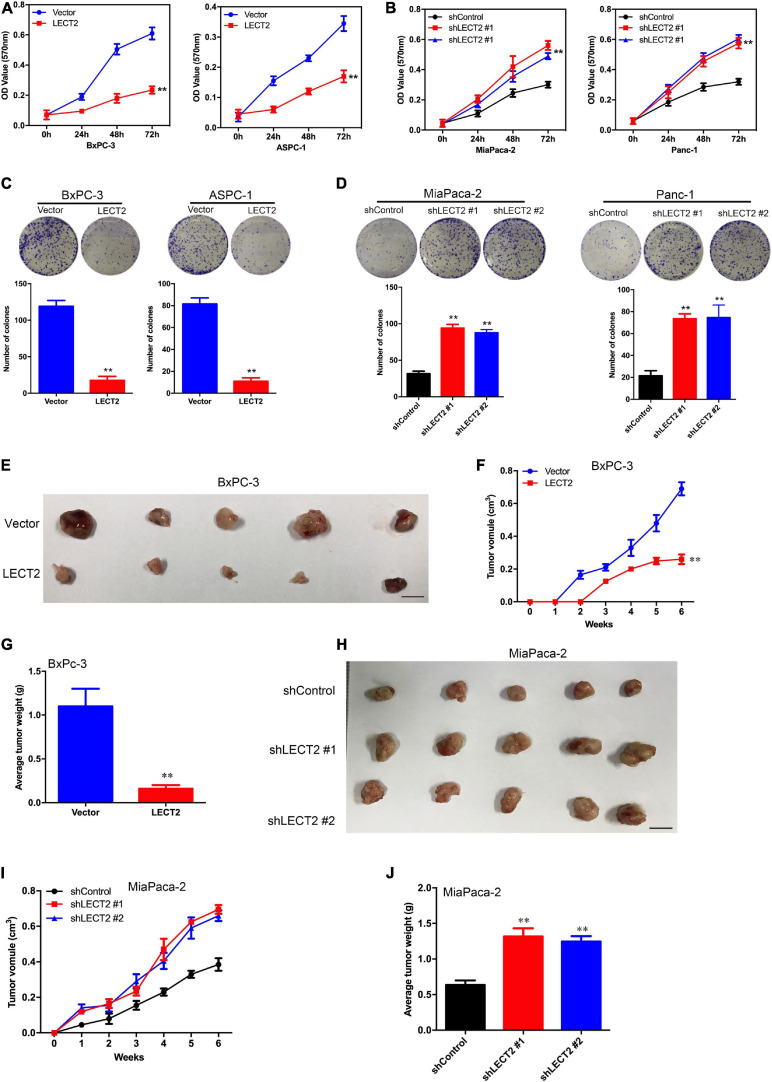
LECT2 regulates cell proliferation, colony formation, and tumor growth in PDAC. **(A)** Cell proliferation of BxPC-3 (left) and ASPC-1 (right panel) cells transfected with control (blue) or LECT2 (red) vector. **(B)** Cell proliferation of MiaPaca-2 (left) and Panc-1 (right panel) cells transfected with shControl (black) or shLECT2 (red and blue) vectors. **(C)** Colony formation of BxPC-3 (left) and ASPC-1 (right panel) cells as described in **(A)**. **(D)** Colony formation of MiaPaca-2 (left) and Panc-1 (right panel) cells as described in **(B)**. **(E–G)** BxPC-3 cells (1 × 10^6^), as described above, were injected subcutaneously into the mice (*n* = 5 for each group), and the resulting tumors in each group were measured once a week. Photographs of tumors isolated from the mice **(E)**, the tumor volume **(F)**, and the weights of the tumors **(G)** are shown. **(H–J)** MiaPaca-2 cells (1 × 10^6^), as described above, were injected subcutaneously into the mice (*n* = 5 for each group), and the resulting tumors in each group were measured once a week. Photographs of tumors isolated from the mice **(H)**, the tumor volume **(I)**, and the weights of the tumors **(J)** are shown. Scale bar = 1 cm in **(E)** and **(H)**. **p < 0.01.

To explore whether LECT2 acts as a tumor suppressor in pancreatic cancer, PDAC cell lines BXPC-3 and ASPC-1 were transfected with lentiviral vectors encoding human LECT2 inserts, whereas MiaPaca-2 and Panc-1 cells were transfected with shRNAs targeting LECT2 based on the endogenous expression of LECT2 (Supplementary Figure 2). The transfection efficiency was confirmed by immunoblotting and RT-qPCR (Supplementary Figure 3). Subsequently, cell viability and colony formation assays were performed to examine the effect of LECT2 on tumor proliferation. As expected, the ectopic expression of LECT2 suppressed cell viability and clonal forming abilities in BXPC-3 and ASPC-1 cells (Figures 2A,C). Conversely, LECT2 deficiency notably promoted these oncogenic behaviors in MiaPaca-2 and Panc-1 cells (Figures 2B,D). To further assess the function of LECT2 in tumor suppression, xenograft mice injected with BXPC-3 cells bearing LECT2 inserts or MiaPaca-2 cells bearing shLECT2 were established, respectively. In agreement with the in vitro findings, xenograft mice injected with BXPC-3 cells displayed reduced tumor volume and weight compared to control models (Figures 2E–G). In contrast, the deficiency of LECT2 in the MiaPaca-2 cells markedly speeded up tumor growth in vivo (Figures 2H–J). Together, these findings indicate that LECT2 detains PDAC tumorigenesis both in vitro and in vivo.

### LECT2 Inhibits Epithelial-Mesenchymal Transition in PDAC

**FIGURE 3:**
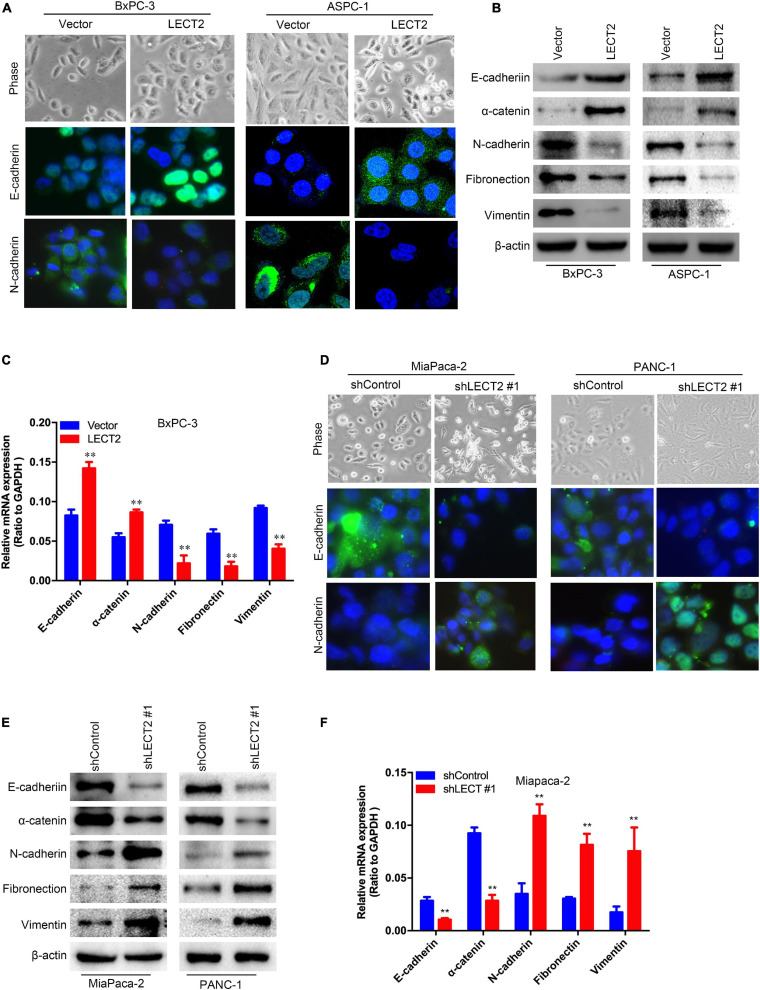
LECT2 inhibits epithelial-mesenchymal transition (EMT) in PDAC. **(A)** Phase-contrast images (top) and immunofluorescence images of transformed BxPC-3 (left) and ASPC-1 (right) cells stained with antibodies against E-cadherin (middle), or N-cadherin (bottom panel). BxPC-3 and ASPC-1 cells were transfected with control or LECT2 vector, as described in Figure 2. **(B)** Immunoblotting analysis of expression of E-cadherin, α-catenin, N-cadherin, Fibronectin, and Vimentin proteins in the cells shown in **(A)**. β-actin was used as a loading control. **(C)** mRNA expression of E-cadherin, α-catenin, N-cadherin, Fibronectin, and Vimentin in transformed BxPC-3 cells as described above. **(D)** Phase-contrast images (top) and immunofluorescence images of transformed MiaPaca-2 (left) and Panc-1 (right) cells stained with antibodies against E-cadherin (middle), or N-cadherin (bottom panel). MiaPaca-2 and Panc-1 cells were transfected with shControl or shLECT2 vector, as described in Figure 2. **(E)** Immunoblotting analysis of expression of E-cadherin, α-catenin, N-cadherin, Fibronectin, and Vimentin proteins in the cells shown in **(D)**. β-actin was used as a loading control. **(F)** mRNA expression of E-cadherin, α-catenin, N-cadherin, Fibronectin, and Vimentin in transformed MiaPaca-2 cells as described above. Scale bar = 20 μm in **(A)** and **(D)**. **p < 0.01.

Since LECT2 expression is downregulated in metastatic PDACs compared with the primary tumor, we speculated that LECT2 might be involved in the regulation of PDAC metastasis. To test the above notion, we first examined the morphology and epithelial-mesenchymal transition (EMT) markers like E-cadherin, N-cadherin, α-catenin, fibronectin, and vimentin in transformed BXPC-3 and ASPC-1 cells (Figures 3A–C and Supplementary Figure 4A). Phase-contrast images clearly showed that BXPC-3 and ASPC-1 cells overexpressing LECT2 displayed epithelial morphology. Consistently, immunofluorescence, immunoblotting, and qPCR analysis indicated high levels of epithelial markers (E-cadherin and α-catenin) and lowered expression of mesenchymal markers (N-cadherin, fibronectin, and vimentin) in LECT2-overexpressing BXPC-3 and ASPC-1 cells. On the other hand, cells devoid of LECT2 manifested the opposite trends (Figures 3D–F and Supplementary Figure 4B). In other words, LECT2-deficient MiaPaca-2 and Panc-1 cells displayed mesenchymal morphology and high levels of mesenchymal markers as well as low levels of epithelial markers. The data mentioned above suggest that LECT2 manages the plasticity between epithelial and mesenchymal states in PDAC cells.

### LECT2 Inhibits PDAC Migration, Invasion, and Distant Lung Metastasis

**FIGURE 4:**
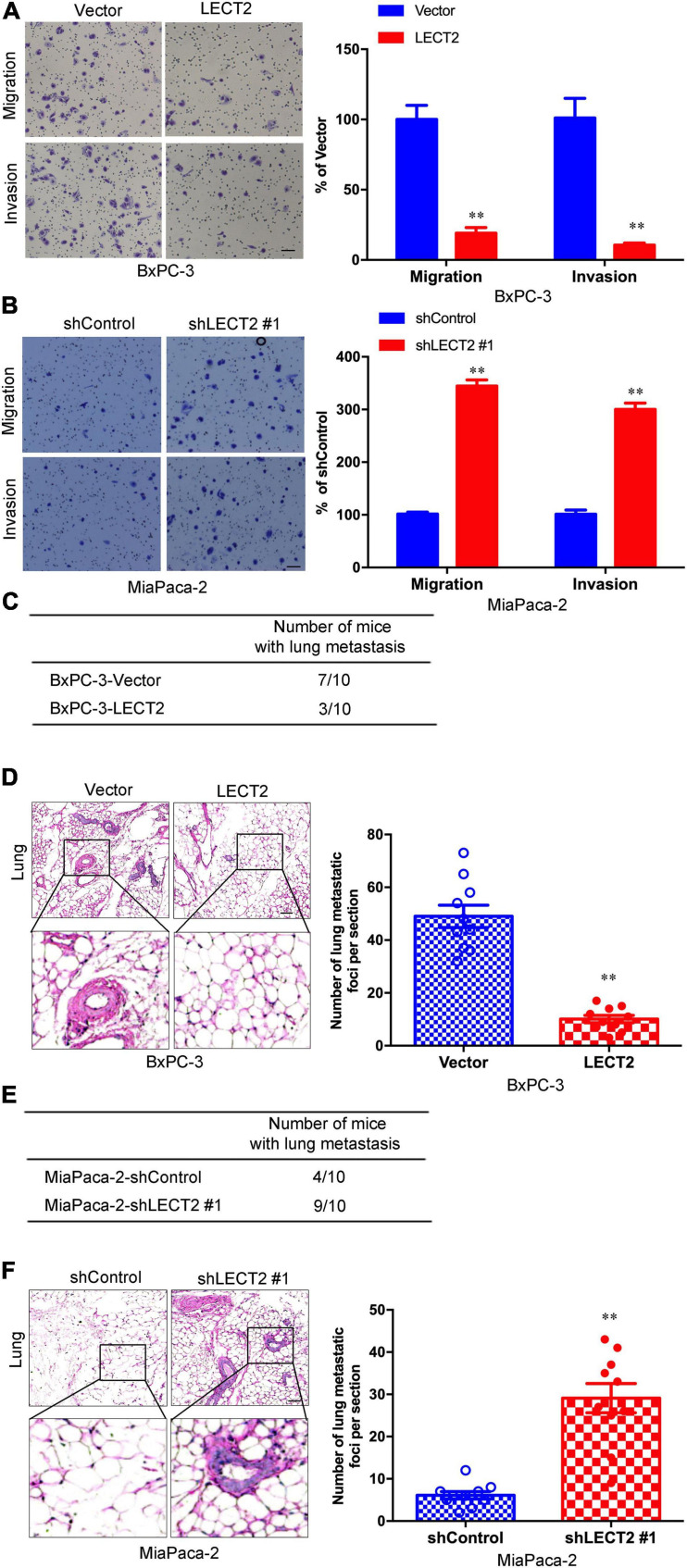
LECT2 inhibits PDAC migration, invasion, and distant lung metastasis. **(A,B)** Representative images of migration (top) and invasion (bottom) of transformed BxPC-3 **(A)** and MiaPaca-2 **(B)** cells described above (left) with corresponding quantifications (right). **(C)** Number of mice with distant lung metastasis following intravenous injections of transformed BxPC-3 cells as described above. **(D)** Representative H&E staining images (left) of metastatic lung foci per section from allograft mice described in **(C)** with corresponding quantifications (right). **(E)** Number of mice with distant lung metastasis following intravenous injections of transformed MiaPaca-2 cells as described above. **(F)** Representative H&E staining images (left) of metastatic lung foci per section from allograft mice described in **(E)** with corresponding quantifications (right). Scale bar = 50 μm in **(A,B)**. Scale bar = 200μm in **(D,E)**. **p < 0.01.

As EMT is considered to be a fundamental process involved in cancer metastasis, the role of LECT2 in migration and invasion was thus investigated. BXPC-3 and ASPC-1 cells with excessive expression of LECT2 were subjected to cell migration and invasion assessment (Figure 4A and Supplementary Figure 5A). As expected, LECT2 overexpression impaired the migratory and invasive abilities of BXPC-3 and ASPC-1 cells. Conversely, ablation of LECT2 promoted migration and invasion in MiaPaca-2 and Panc-1 cells (Figure 4B and Supplementary Figure 5B). These findings together indicated that LECT2 inhibited migration and invasion in PDAC cells. To further explore the effect of LECT2 on metastasis in a physiologic tumor context, we developed a xenograft model, employing BXPC-3 cells expressing LECT2 vectors. In line with in vitro results, LECT2 overexpression reduced considerably the number of mice with lung metastasis (7 of 10, BxPC-3-Vector; 3 of 10, BxPC-3-LECT2), and the number of metastatic lung foci per section as shown by the H&E staining of lung tissues from mice after intravenous injection (Figures 4C,D). Meanwhile, LECT2 knockdown increased those numbers compared to control (4 of 10, MiaPaca-2-shControl; 9 of 10, MiaPaca-2-shLECT2#1) (Figures 4E,F). Hence, LECT2 regulates not only EMT but also regulates both migration and invasion of PDAC cells, consequently influencing in vivo metastasis.

### LECT2 Negatively Correlates With Forkhead Box M1 Signaling Pathway and Downregulates FOXM1 Expression

**FIGURE 5:**
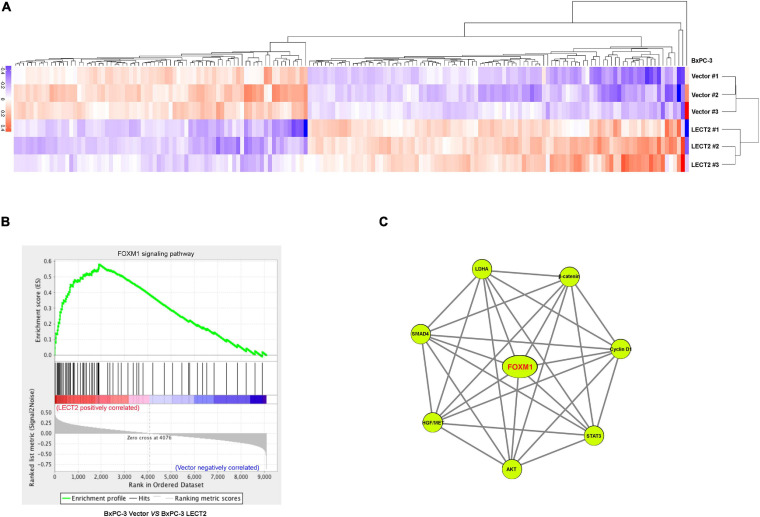
LECT2 negatively correlates with the FOXM1 signaling pathway. **(A)** Heatmap summarizing genes that differentially expressed in BxPC-3 cells transfected with control or LECT2 vectors. Up-regulated genes were labeled in red, and downregulated genes were shown in purple. **(B)** Enrichment of the FOXM1 signaling pathway in Gene Set Enrichment Analysis (GSEA) of genes altered as described above. **(C)** Protein-Protein-Interaction Network, including the enriched genes in the FOXM1 signaling pathway.

Next sought to explore how LECT2 regulates the tumorigenesis and metastasis of PDAC. To decipher the underlying mechanism of PDAC development and progression impeded by LECT2, we performed RNA-seq with BXPC-3 cells bearing empty vectors or LECT2 inserts (Figure 5A). The RNA-seq results showed that there were 215 up-regulated genes and 173 down-regulated genes (Supplemental Table 1). Gene set enrichment analysis (GSEA) revealed the forkhead box M1 (FOXM1) signaling pathway as the enriched signature reversely correlated with endogenous LECT2-dependent transcription in PDAC (Figure 5B). Protein-Protein-Interaction Network further showed the enriched genes in the FOXM1 signaling pathway (Figure 5C).

**FIGURE 6:**
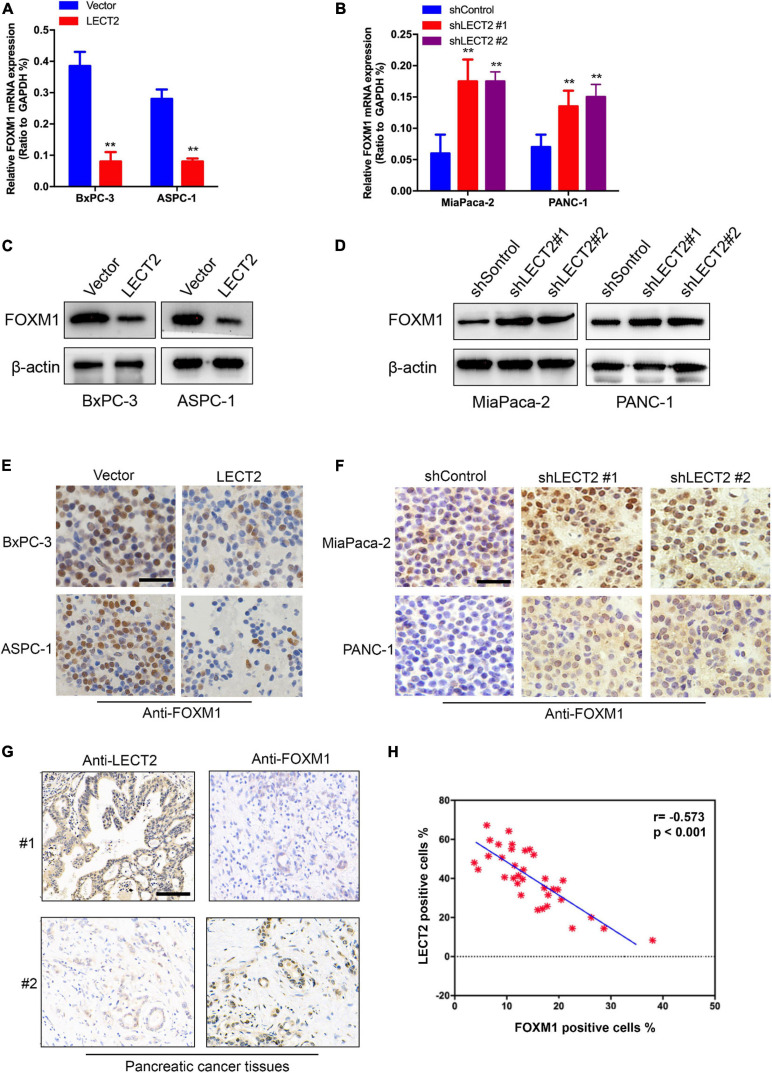
LECT2 downregulates FOXM1 expression. **(A)** mRNA expression of FOXM1 in BxPC-3 and ASPC-1 cells transfected with control (blue) or LECT2 (red) vector. **(B)** mRNA expression of FOXM1 in MiaPaca-2 and Panc-1 cells transfected with shControl (blue) or shLECT2 (red and magenta) vector. **(C)** Immunoblotting to measure FOXM1 protein levels in BxPC-3 (left) and ASPC-1 (right) cells described in **(A)**. **(D)** Immunoblotting to measure FOXM1 protein levels in MiaPaca-2 (left) and Panc-1 (right) cells described in **(B)**. **(E)** Representative IHC staining images of FOXM1 in BxPC-3 (top) and ASPC-1 (bottom) cells described in **(A)**. **(F)** Representative IHC staining images of FOXM1 protein levels in MiaPaca-2 (top) and Panc-1 (bottom) cells described in **(B)**. **(G)** Representative IHC staining images of LECT2 and FOXM1 in pancreatic cancer tissues from 2 patients. **(H)** Quantification of **(G)**. Scale bar = 400 μm in **(E,F)**. Scale bar = 200 μm in **(G)**. **p < 0.01.

To investigate LECT2’s effect on FOXM1 expression, qRT-PCR and immunoblotting were applied to total RNA and protein extracted from BXPC-3 and ASPC-1 cells with excessive expression of LECT2 or LECT2-deficient MiaPaca-2 and Panc-1 cells (Figures 6A–D). In BXPC-3 and ASPC-1 cells, LECT2 overexpression led to lower levels of FOXM1 and its downstream target genes, such as cyclin D1, cyclin B1, and c-Myc (Figures 6A,C, and Supplementary Figure 6A). Likewise, the depletion of LECT2 resulted in enhanced expression of FOXM1 and its downstream target genes in MiaPaca-2 and Panc-1 cells (Figures 6B,D, and Supplementary Figure 6B). These findings were further supported by the immunochemistry evidence from transformed PDAC cells and pancreatic cancer tissues showing the inverse correlation between LECT2 and FOXM1 (Figures 6E–H). Also, we found that overexpression of FOXW1 significantly increased the cell viability, clone formation ability, and invasion/migration ability of BxPC-3 cells (Supplementary Figure 7). Collectively, these findings suggested that LECT2 negatively mediated the FOXM1 signaling pathway in PDAC.

### FOXM1 Is Necessary for the Suppressive Impact of LECT2 on PDAC Malignancy

**FIGURE 7:**
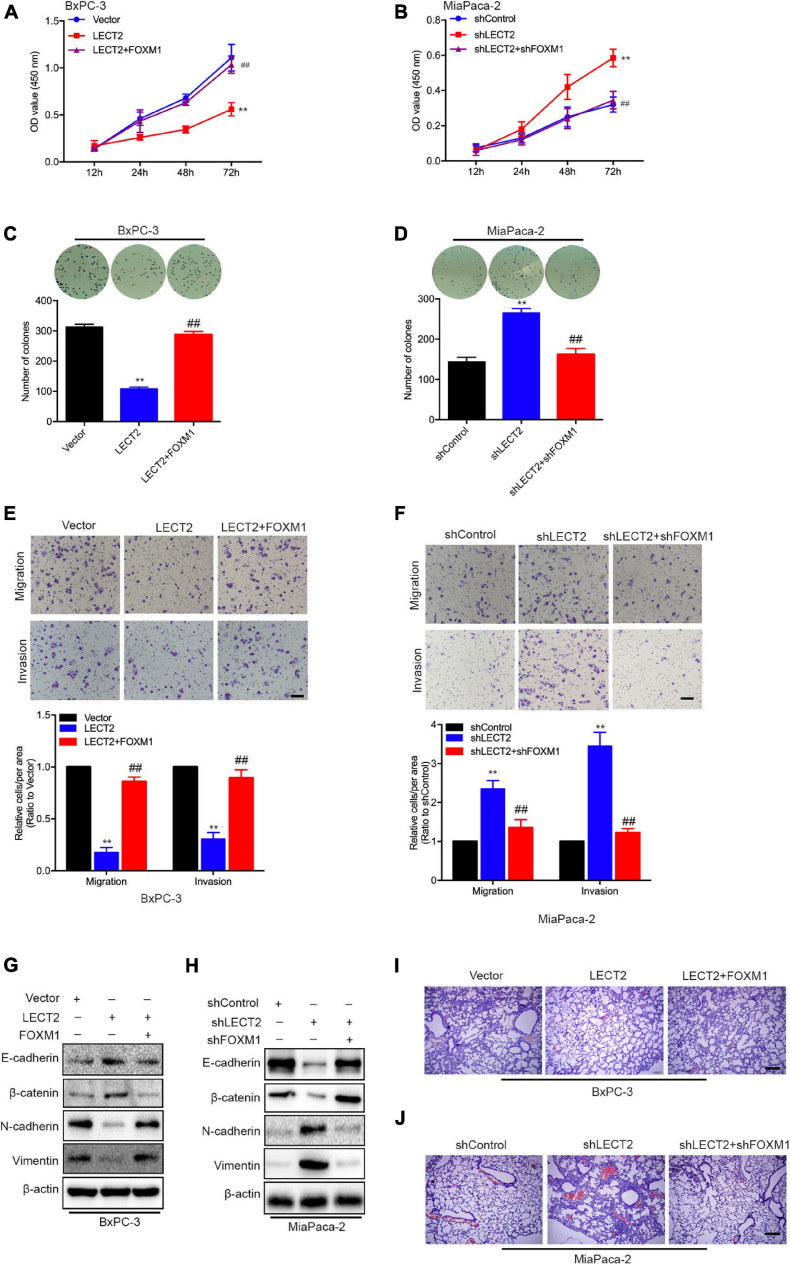
LECT2 suppresses malignant behaviors depending on FOXM1. **(A)** Cell proliferation of BxPC-3 cells transfected with control (blue), LECT2 (red), or LECT2 together with FOXM1 (magenta) vectors. **(B)** Cell proliferation of MiaPaca-2 cells transfected with shControl (blue), shLECT2 (red), or shLECT2 together with shFOXM1 (magenta) vectors. **(C,D)** Colony formation of BxPC-3 **(C)** and MiaPaca-2 **(D)** cells as described in **(A,B)**. **(E,F)** Migration (top) and invasion (middle) of BxPC-3 **(E)** and MiaPaca-2 **(F)** cells as described above with corresponding quantifications (bottom). **(G,H)** Immunoblotting analysis of E-cadherin, β-catenin, N-cadherin, and Vimentin protein levels in BxPC-3 **(G)** and MiaPaca-2 **(H)** cells as described above. **(I,J)** Representative H&E staining images of metastatic lung foci per section from allograft mice injected with BxPC-3 **(I)** and MiaPaca-2 **(J)** cells as described above. Scale bar = 50 μm in **(E,F)**. Scale bar = 200 μm in **(I,J)**. **p < 0.01 and ^##^*p* < 0.01.

To prove whether FOXM1 is required for the inhibitory effect of LECT2 on PDAC malignancy, we established two stable cell lines, BXPC-3 cells expressing LECT2 as well as FOXM1 insert, and MiaPaca-2 cells transfected with shRNAs that target LECT2 as well as FOXM1. Subsequently, cell viability and colony formation assays were performed to examine the involvement of FOXM1 in the effect of LECT2 on tumor proliferation (Figures 7A–D). As anticipated, the ectopic expression of FOXM1 in BXPC-3 cells almost completely abolished the inhibitory effect of LECT2 overexpression on cell viability and colony formation (Figures 7A,C). In contrast, FOXM1 deficiency dramatically reduced the impact of LECT2 knockdown on cell viability and sphere formation in MiaPaca-2 cells (Figures 7B,D). Similarly, migration and invasion assay showed that the reintroduction of FOXM1 restored the aggressive abilities of BXPC-3 cells undermined by LECT2 overexpression (Figure 7E). Besides, we found that the promoting effect of LECT2 deficiency on cell migration and invasion was impaired when FOXM1 was also knocked down (Figure 7F). Since LECT2 mediated the transition between epithelial and mesenchymal states, immunoblotting was thus applied to evaluate the function of FOXM1 on EMT markers (Figures 7G,H). The data showed that epithelial phenotype favored by excessive expression of LECT2 was revoked by FOXM1 the overexpression in BxPC-3 cells, whereas FOXM1 knockdown abolished the mesenchymal states induced by LECT2 deficiency in MiaPaca-2 cells.

Most Importantly, in vivo evidence from mouse xenografts injected with the transformed BxPC-3 orMiaPaca-2 cells revealed that reintroduction of FOXM1 reversed the inhibitory effects of LECT2 on distant metastasis. In contrast, the ablation of FOXM1 attenuated the lung metastasis induced by LECT2 deficiency (Figures 7I,J). The results, as mentioned above, demonstrated that LECT2 might suppress the malignant behaviors of PDAC cells via downregulating FOXM1 expression.

### Hepatocyte Growth Factor/MET Is Involved in the Regulation of FOXM1 by LECT2

**FIGURE 8:**
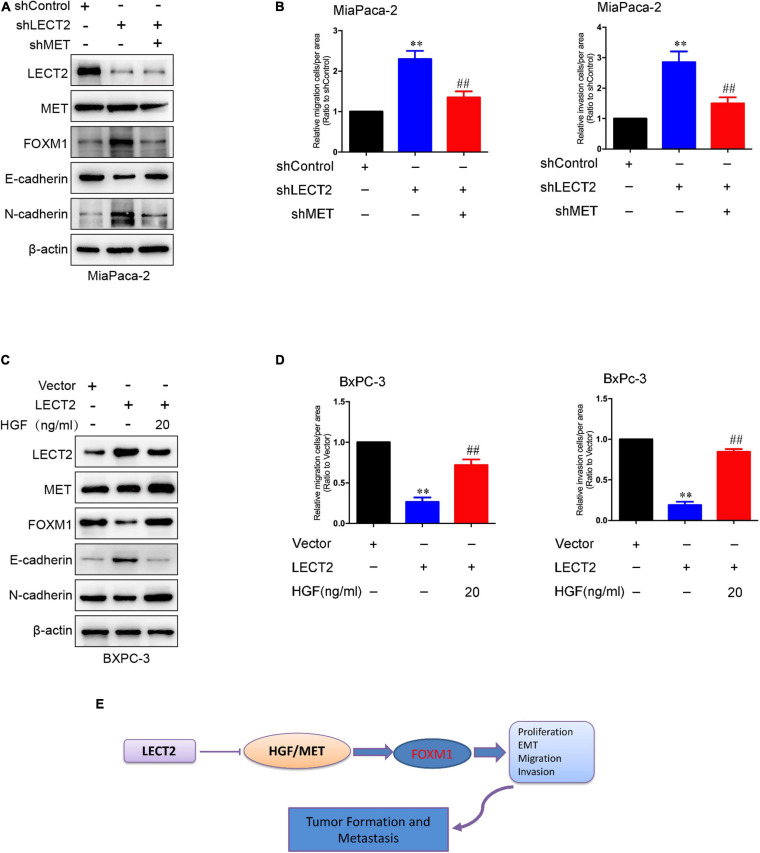
HGF/MET is involved in the regulation of FOXM1 by LECT2. **(A)** Immunoblotting analysis of LECT2, MET, FOXM1, E-cadherin, and N-cadherin protein levels in MiaPaca-2 cells transfected with shControl, shLECT2, and shMET. **(B)** Migration (left) and invasion (right) of MiaPaca-2 cells described in **(A)**. **(C)** Immunoblotting analysis of LECT2, MET, FOXM1, E-cadherin, and N-cadherin protein levels in BxPC-3 cells transfected with Control, LECT2, and/or HGF vectors. **(D)** Migration (left) and invasion (right) of BxPC-3 cells described in **(C)**. **(E)** Schematic model illustrating the biological processes regulated by LECT2 in PDAC. **p < 0.01 and ^##^*p* < 0.01.

The co-presence of both MET and FOXM1 is common in patients with gastric cancer (Francica et al., 2016). Besides, a positive feedback loop between FOXM1 and HGF/MET has been identified in PDAC, which contributes to PDAC growth (Cui et al., 2016). Since LECT2 was reported to antagonize MET receptor activation in hepatocellular carcinoma and non-small cell lung cancer (Chen et al., 2014; Hung et al., 2018), we speculated that LECT2 might regulate FOXM1 signaling by targeting MET. To test the notion mentioned above, two stable cell lines were established, MiaPaca-2 cells transfected with shRNAs that target LECT2 as well as MET, and BXPC-3 cells expressing LECT2, as well as MET, insert. Immunoblotting, migration, and invasion assays were performed, respectively, to measure the function of MET on LECT2 affected malignant behaviors (Figure 8). As expected, MET knockdown had a slight effect on LECT2 but completely blocked the expression of FOXM1 (Figure 8A). Additionally, coincident observations were achieved by immunoblotting together with migration and invasion assay that MET deficiency completely abrogated the promoting effects of LECT2 loss on these malignant behaviors in MiaPaca-2 cells (Figures 8A,B). However, knocking out LECT2 did not affect MET protein levels. Therefore, we also tested the phosphorylation level of MET after knockout or overexpression of LECT2. The experimental results showed that knocking out LECT2 significantly increased the expression level of p-MET. At the same time, the overexpression of LECT2 inhibited the expression level of p-MET (Supplementary Figures 8A,B). Furthermore, a MET inhibitor (Selumetinib) was used to treat MiaPaca-2 cells knocked out of LECT2. The experimental results showed that the effect of knocking out LECT2 on the increase of cell migration and invasion was reversed (Supplementary Figures 8C,D). On the other hand, HGF partially recovered the FOXM1 expression and the migratory and invasive abilities in BXPC-3 cells expressing LECT2 (Figures 8C,D). Overall, these observations indicated that LECT2 might antagonize FOXM1 signaling via targeting HGF/MET, which affects multiple biological processes and slow down PDAC tumor formation and metastasis (Figure 8E). Finally, we also stably overexpress HGF in BxPC-3 cells overexpressing LECT2 (Supplementary Figure 8E). BxPC-3 cells co-overexpressing LECT2 and HGF reversed the inhibitory effect of LECT2 on cell invasion and migration (Supplementary Figures 8F,G). These results indicate that LECT2 inhibits FOXM1 by negatively regulating HGF/MET, leading to an inhibitory effect on the metastasis of pancreatic cancer cells.

## Discussion

The incidence of PDAC is steadily increasing, with the highest mortality-to-incidence ratio amongst all solid organ cancers (Mizrahi et al., 2020). However, the diagnosis and prognosis are still miserable, highlighting the necessity of novel biomarkers and therapeutic targets. However, accumulated evidence showed that the clinical implications of LECT2 in various malignancies, the molecular mechanisms of LECT2 in PDAC progression remain mostly unclear. In this study, we showed that LECT2 serves as a tumor suppressor in PDAC progression as follows: (1) LECT2 is downregulated in metastatic PDACs compared with the primary tumor, and its expression is negatively correlated with multiple clinical pathologic features and prognosis. (2) Overexpression of LECT2 attenuates PDAC tumor growth and metastasis in vitro and in vivo. (3) Mechanistically, LECT2 inhibits FOXM1 signaling by targeting HGF/MET, which retards PDAC progression.

To explore whether LECT2 is involved in PDAC development, we first detected LECT2 expression in cell lines and tissues from PDAC patients. LECT2 expression significantly decreased in PDACs, especially the metastatic tumors, indicating the potential suppressive role of LECT2 in this malignancy. Moreover, by using PDAC cell lines bearing LECT2 inserts or shRNAs targeting this protein, as well as the corresponding xenografts injected with the transformed cells, we demonstrated that LECT2 overexpression markedly alleviated cell proliferation, EMT, migration, and invasion. In contrast, ablation of LECT2 had the opposite effects. These results suggest the potential application of LECT2 as a promising biomarker or clinical therapeutic target for the diagnosis and treatment of PDAC in the future.

FOXM1 is a member of the fork headbox (Fox) protein superfamily, which is characterized by a conserved winged-helix DNA-binding domain (Clark et al., 1993). As a critical proliferation-associated transcription factor, FOXM1 is a master factor involved in cell cycle transition and directly or indirectly transactivates the expression of target genes that are strictly involved in the processes of cell proliferation, self-renewal, and tumorigenesis (Wierstra, 2013; Laissue, 2019). Increased expression of FOXM1 is identified in multiple human cancers, including ovarian cancer, breast cancer, prostate cancer, hepatocellular carcinoma, colorectal cancer, melanoma, lung cancer, and gastric cancer, which indicates a poor prognosis in most solid tumors. Meanwhile, inhibition of FOXM1 in cancer cells represses cell proliferation, EMT, migration, metastasis, angiogenesis, and drug resistance (Liao et al., 2018). Given its explicit oncogenic nature, FOXM1 has emerged as a promising target for cancer treatment (Xu et al., 2015). However, drugs targeting FOXM1 have yet to be fully explored, which is likely due to the poor current understanding of the mechanism underlying FOXM1 dysregulation. Here, we discovered that LECT2 negatively correlates with the FOXM1 signaling pathway and downregulates FOXM1 expression and its downstream target genes, which contributed to the suppressive impact of LECT2 on PDAC malignancy. Considering the positive feedback loop between FOXM1 and HGF/MET in PDAC, the implication of HGF/MET in the mediation of FOXM1 by LECT2 was also assessed, which may facilitate a comprehensive understanding of FOXM1 regulation in solid tumor.

In conclusion, the current study provides validating evidence that LECT2 acts as a tumor suppressor in PDAC, and the expression of LECT2 in PDAC cells and tissue is significantly lower, which predicts reduced survival. LECT2 significantly inhibits the viability, colony formation, migration, and invasion of PDAC cells in vitro. It retards tumor growth and metastasis in vivo. On the mechanic level, FOXM1 signaling is critical for the inhibitory effects of LECT2 on the malignant behaviors in PDAC. LECT2 regulates FOXM1 expression through targeting HGF/MET. LECT2 could thus serve as a promising biomarker and target for PDAC prognosis and treatment.

## Data Availability Statement

The datasets presented in this study can be found in online repositories. The names of the repository/repositories and accession number(s) can be found below: NCBI GEO; GSE168611.

## Ethics Statement

The studies involving human participants were reviewed and approved by the cancer tissue used complies with the regulations of the Ethics Committee of Qingdao University. The patients/participants provided their written informed consent to participate in this study. The animal study was reviewed and approved by the mice used complied with the rules of the Qingdao University Ethics Committee on the correct use of animals.

## Author Contributions

XL and PL contributed equally to this work and completed relevant experiments. YT, XJ, and TL completed part of the experiment and processed part of the experimental data. YW completed the revision of the manuscript. CW and YC designed the work and preparation of the manuscript. All authors contributed to the article and approved the submitted version.

## Conflict of Interest

The authors declare that the research was conducted in the absence of any commercial or financial relationships that could be construed as a potential conflict of interest.
