# Supplementary material for: LECT 2 Antagonizes FOXM1 Signaling via Inhibiting MET to Retard PDAC Progression
Source: Front Cell Dev Biol. 2021 Apr 15;9:661122. doi: 10.3389/fcell.2021.661122 (PMC8082113; doi:10.3389/fcell.2021.661122)
Supplement: Supplementary file 1 [file Table_1.DOCX]

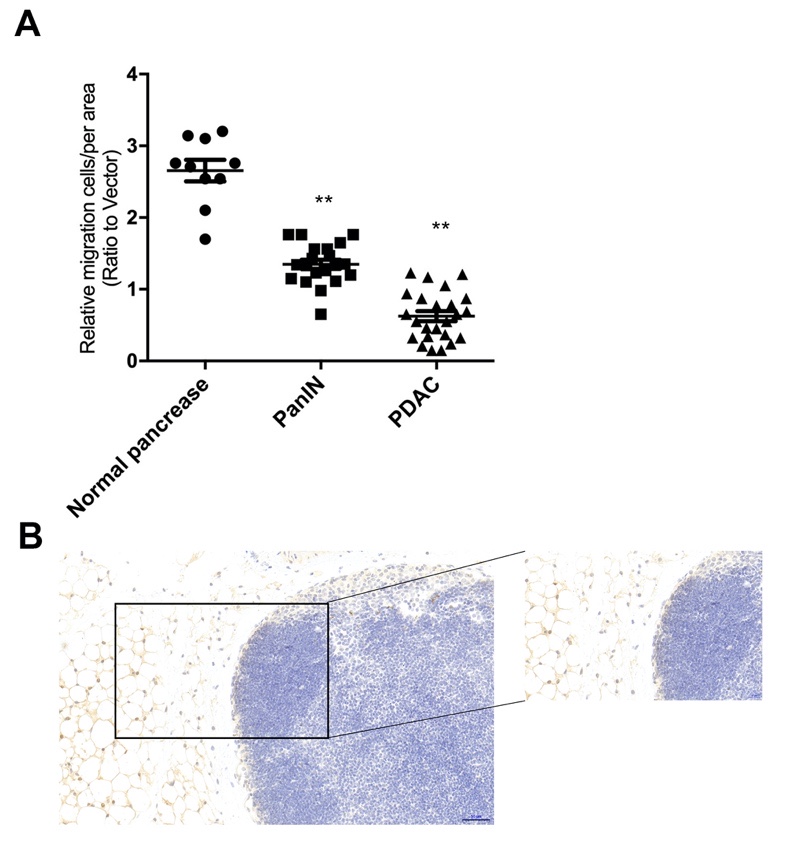


Supplementary Fig 1 (A) Relative migration cells/per area of the normal pancreas, PanIN, and PDAC. (B) The expression level of LECT2 in the tumor and adjacent areas of the tumor. The scale bar in B is 50 μm.


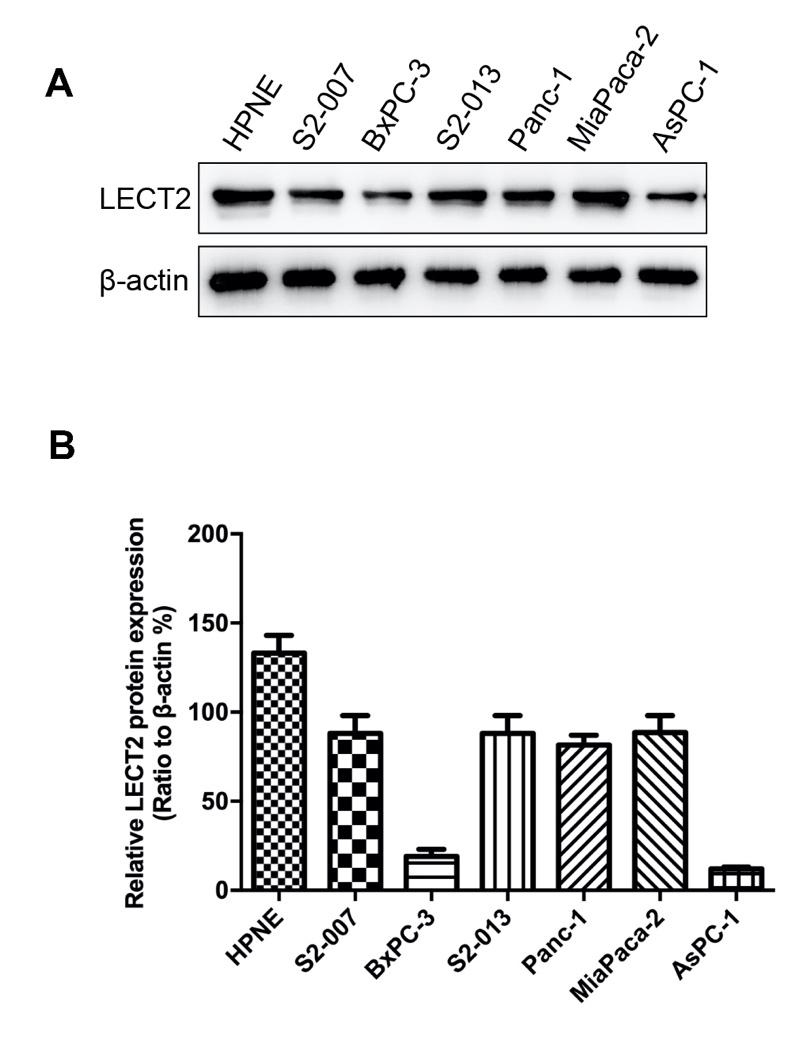


Supplementary Fig 2 LECT2 protein levels in PDAC cell lines (A) and its quantification (B).


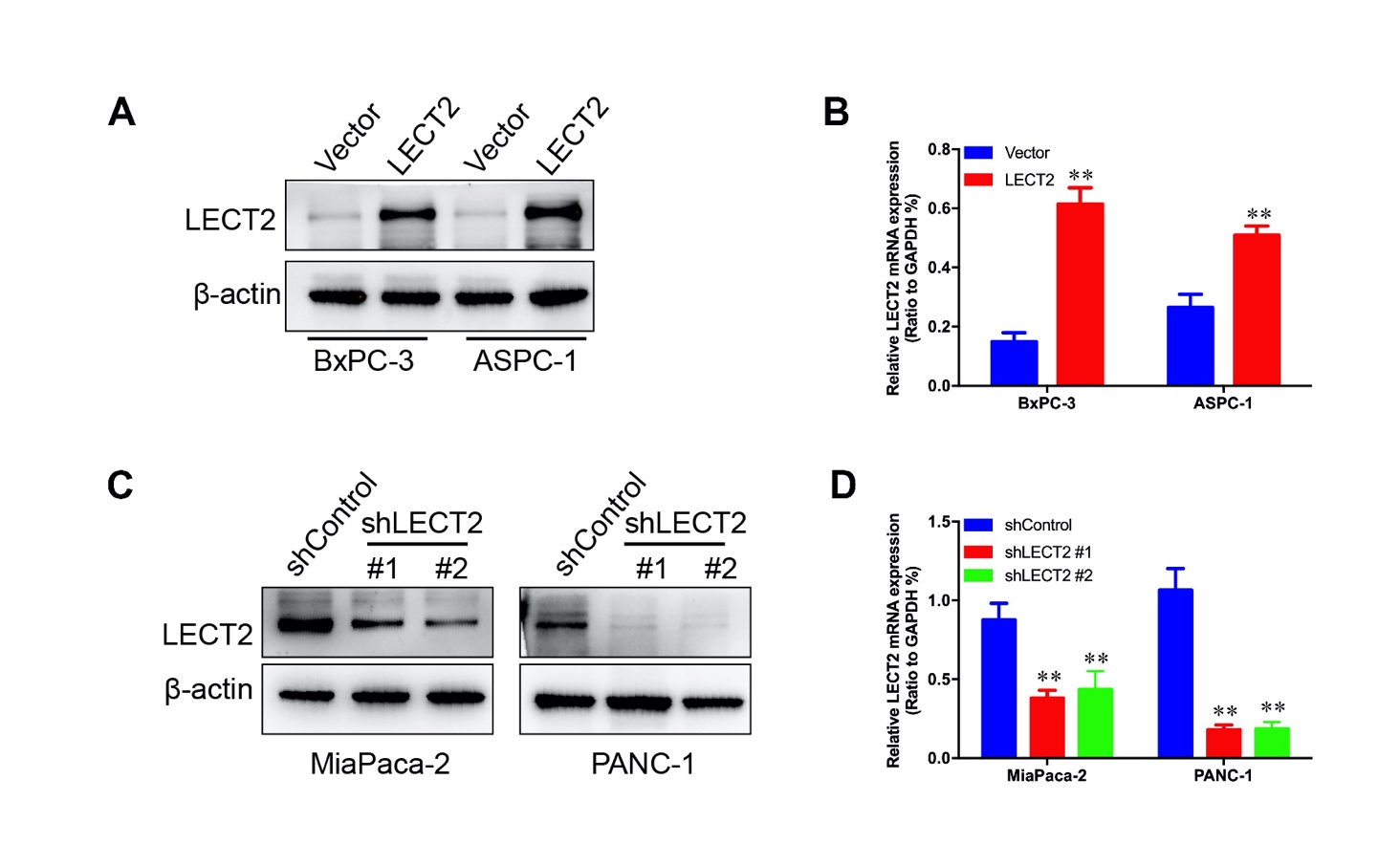


Supplementary Fig 3 (A, B) Immunoblotting (A) and mRNA expression (B) of LECT2 in BxPC-3 and ASPC-1 cells described in Fig.2A. (C, D) Immunoblotting (C) and mRNA expression (D) of LECT2 in MiaPaca-2 and Panc-1 cells described in Fig.2B.


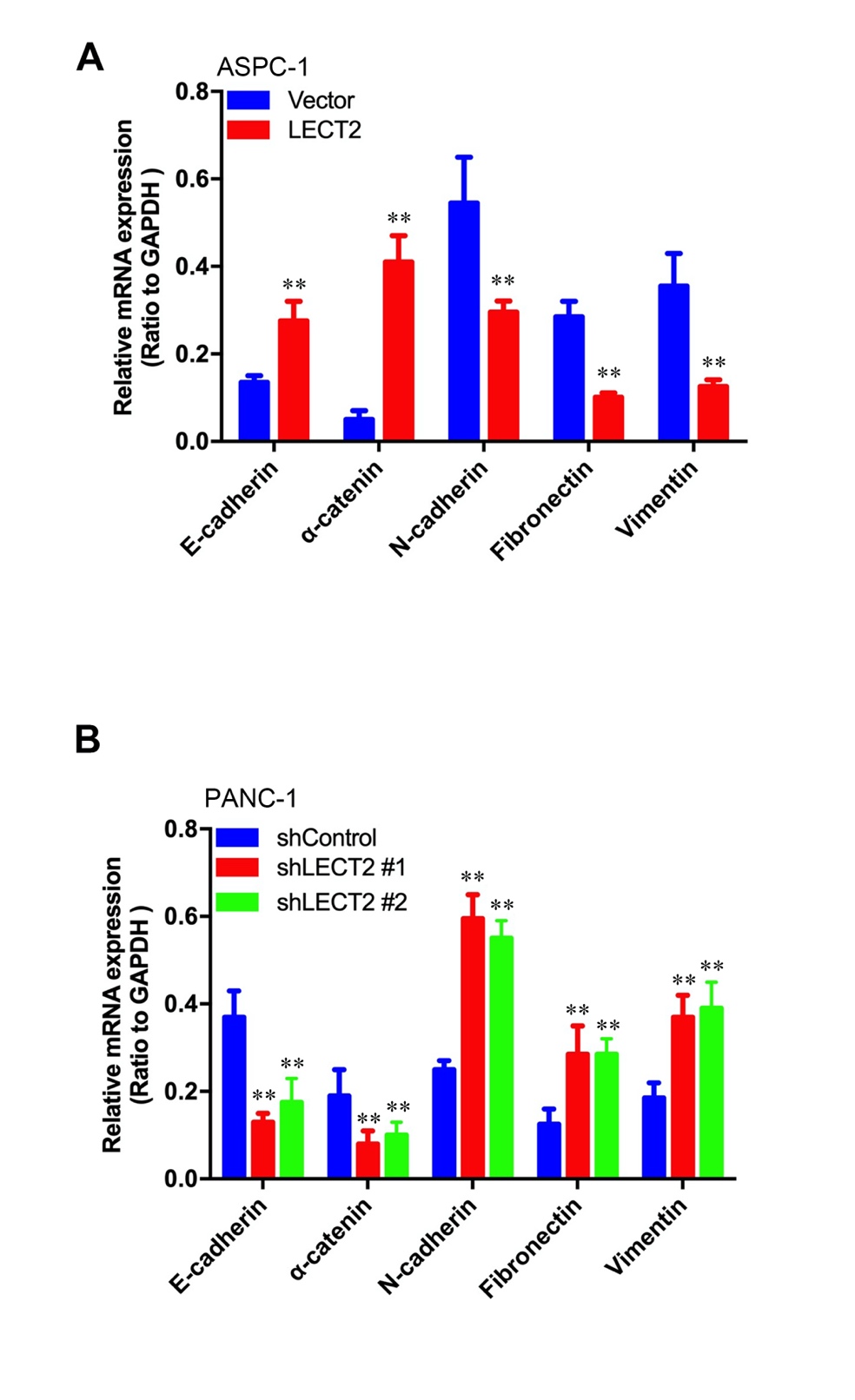


Supplementary Fig 4 mRNA expression of E-cadherin, α-catenin, N-cadherin, Fibronectin, and Vimentin in transformed ASPC-1 (A) and Panc-1 (B) cells as described in Fig.3.


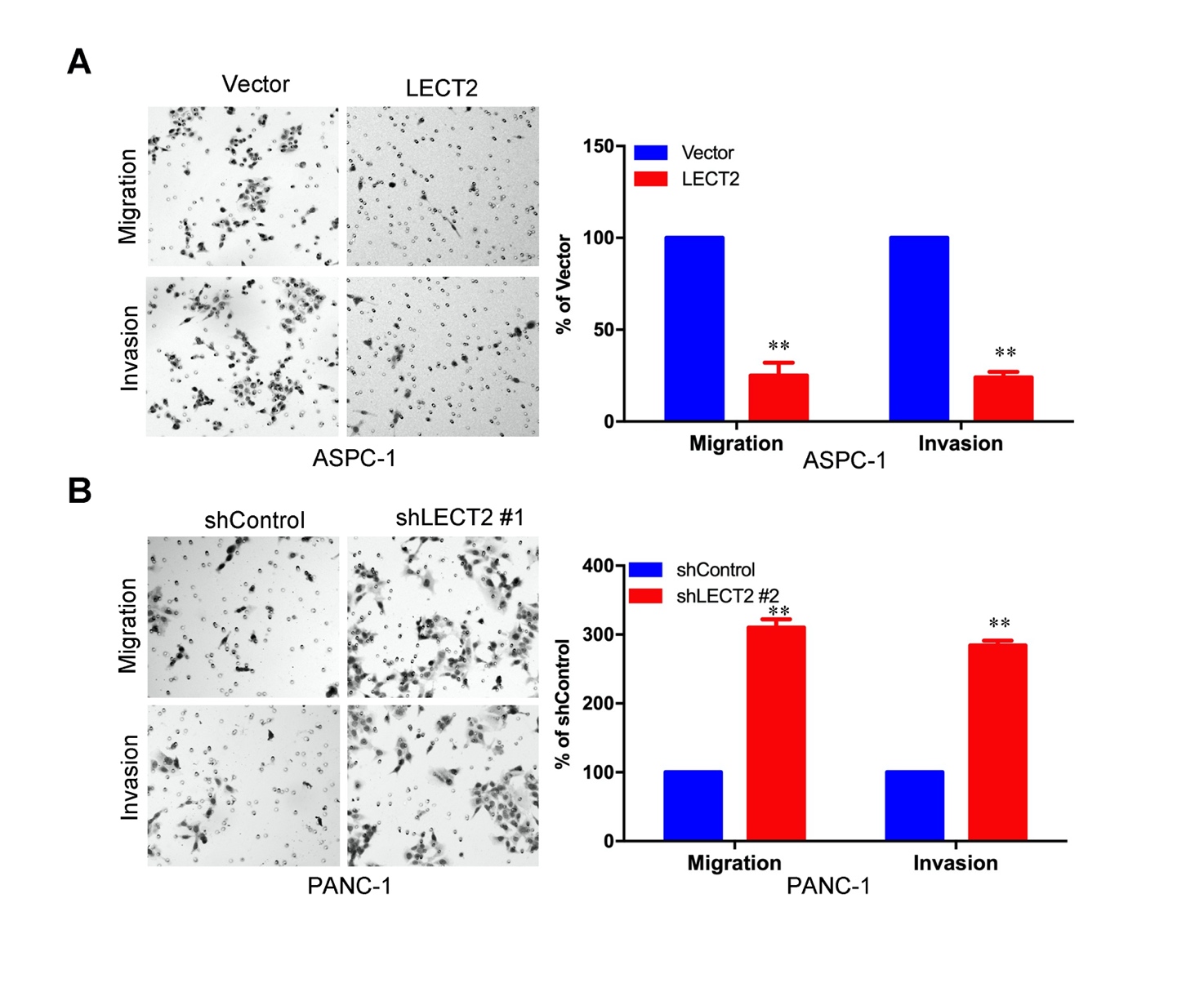


Supplementary Fig 5 Migration (top) and invasion (bottom) of ASPC-1 (A) and Panc-1 (B) cells, as described in Fig.3.


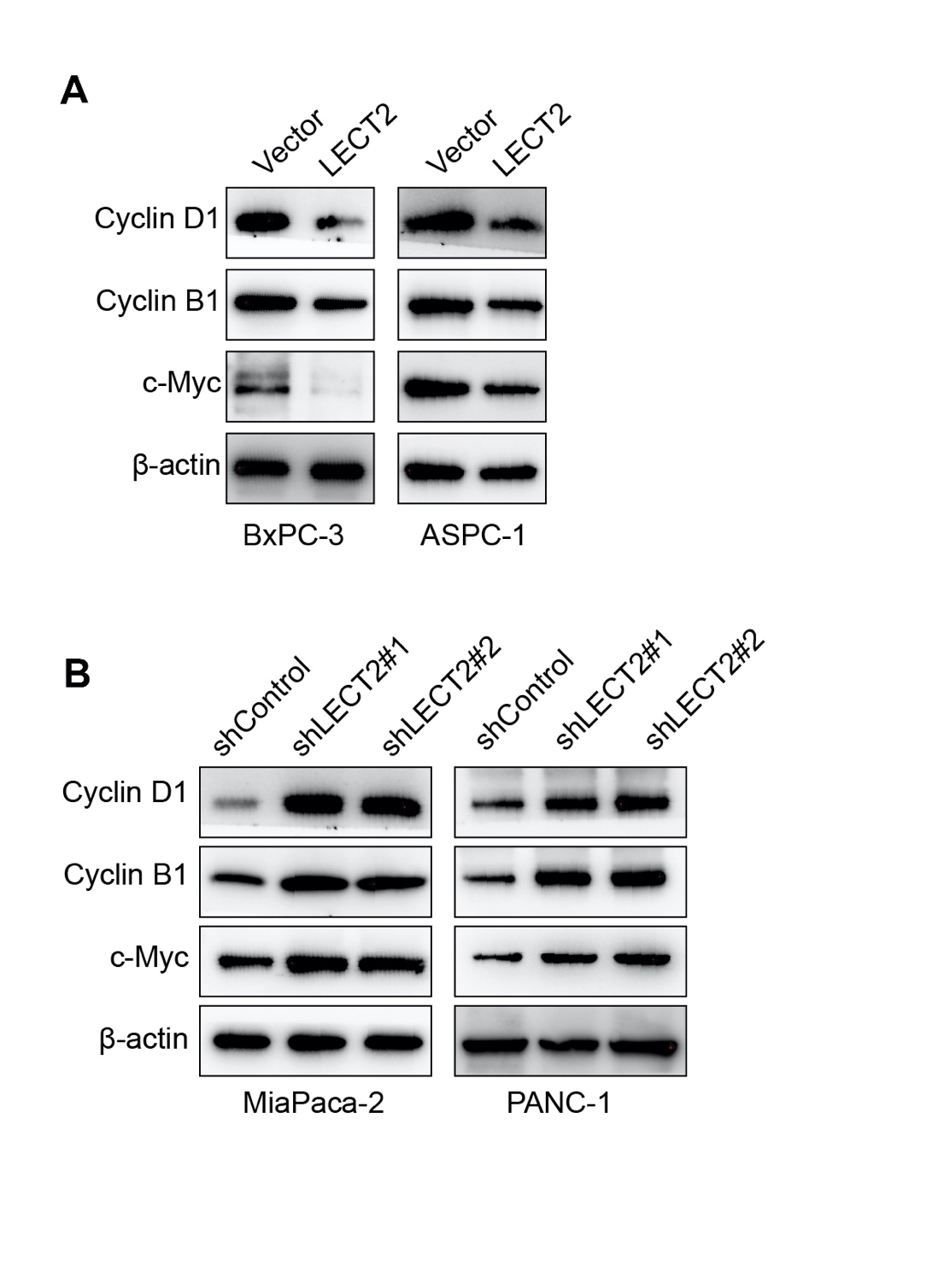


Supplementary Fig 6 Immunoblotting of Cyclin D1, Cyclin B1, and c-Myc proteins in transformed BxPC-3 and ASPC-1 cells (A) and MiaPaca-2 and Panc-1 cells (B) as described in Fig.3.


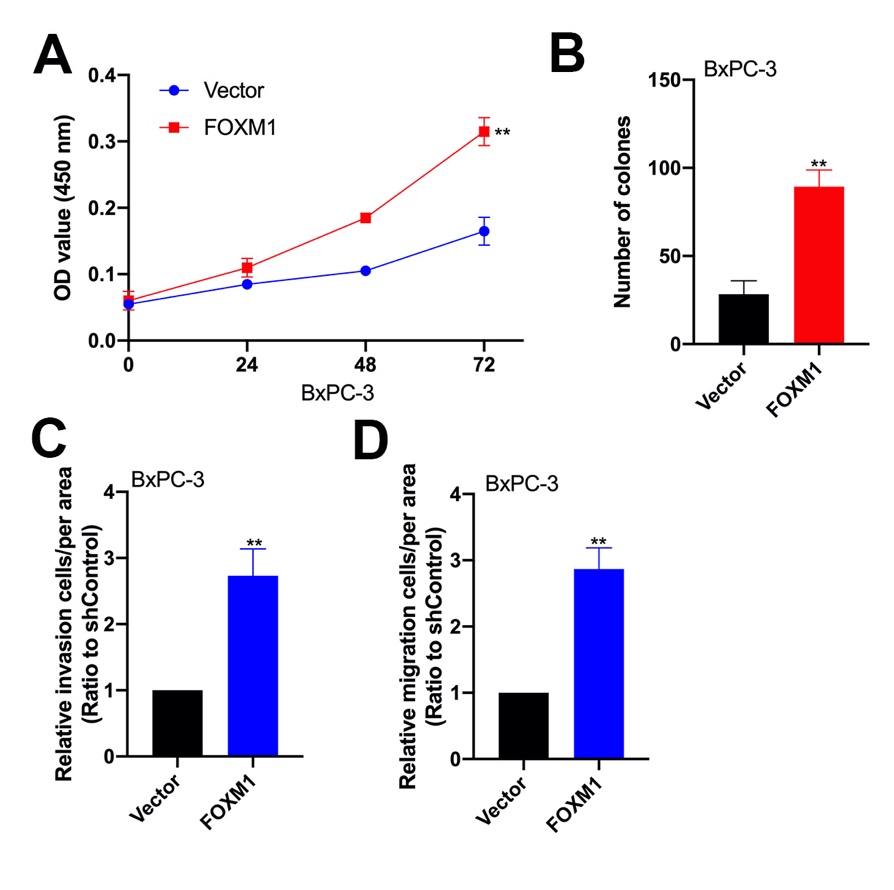


Supplementary Fig 7 BxP-3 cells overexpressing FOXW1 were tested for cell viability (A), clone formation ability (B), and invasion/migration ability (C, D).


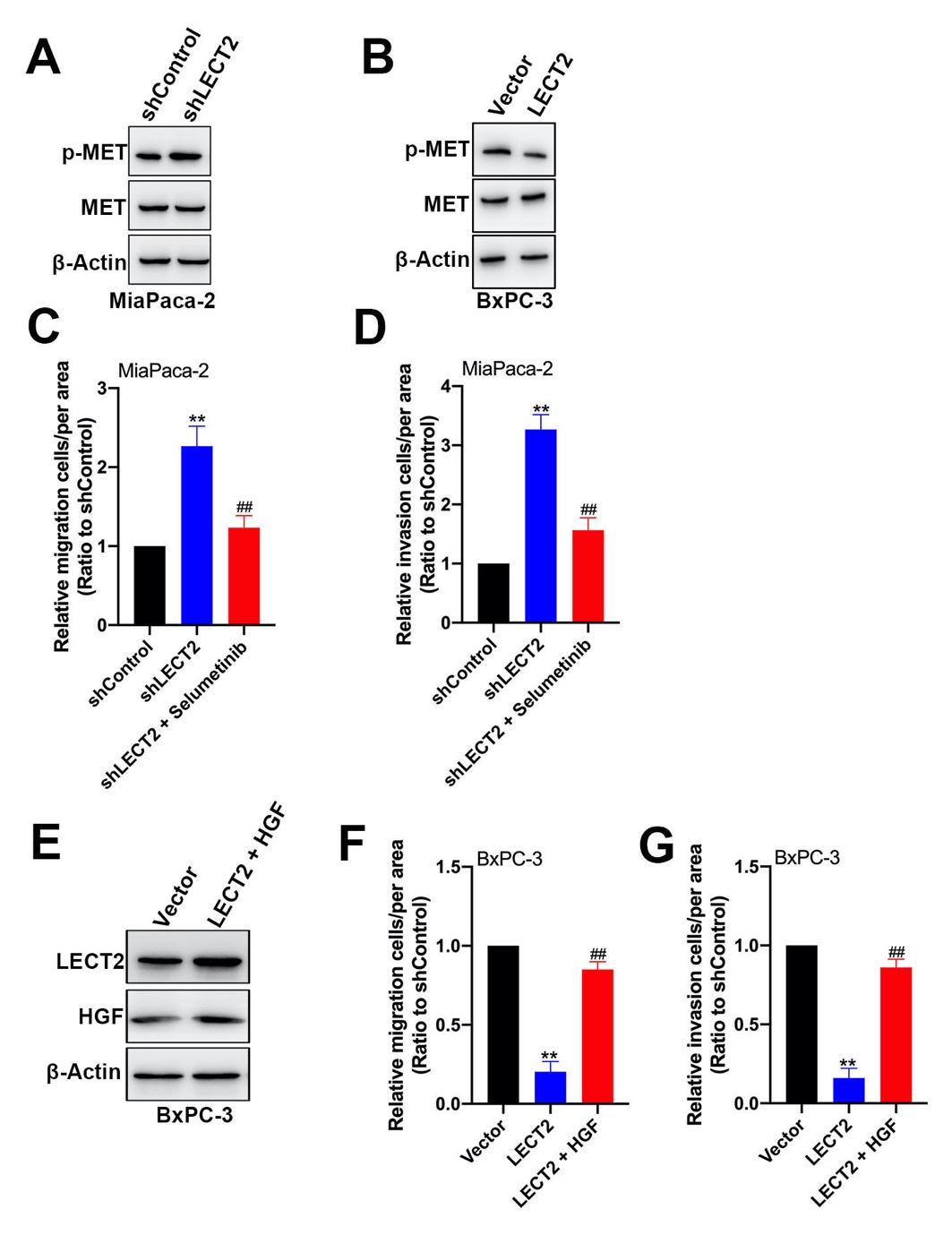


Supplementary Fig 8 (A) The expression level of p-MET in MiaPaca-2 cells with LECT2 knocked out. (B) The expression level of p-MET in BxPC-3 cells overexpressing LECT2. (C, D) MET inhibitor (Selumetinib) was treated with MiaPaca-2 cells knocked out of LECGT2 to detect the invasion and migration ability. (E) Overexpression of LECT2 and HGF in BxPC-3 cells. (F, G) Invasion and migration were detected in BxPC-3 cells overexpressing LECT2 and HGF.

Supplement table 1

RNA-seq results showed a list of down-regulated genes (173) in BxPC-3 cells overexpression LECT2

| Symbol | logFC | test_stat | p_value | adj.P.Val |
| --- | --- | --- | --- | --- |
| FOXM1 | -10.3387 | -13.9036 | 5.00E-05 | 0.00014239 |
| XAF1 | -9.8481 | -3.90271 | 0.0002 | 0.00264676 |
| MX2 | -9.7605 | -8.38136 | 5.00E-05 | 0.00014239 |
| MX1 | -8.9435 | -22.4013 | 5.00E-05 | 0.00014239 |
| TRIM22 | -8.8949 | -9.6294 | 0.00565 | 0.0575853 |
| MX2 | -8.22691 | -15.9058 | 5.00E-05 | 0.00014239 |
| MX2 | -8.11003 | -16.6052 | 5.00E-05 | 0.00014239 |
| CMPK2 | -7.6489 | -10.8808 | 5.00E-05 | 0.00014239 |
| MX2 | -7.58736 | -14.7769 | 5.00E-05 | 0.00014239 |
| N-cadherin | -7.57838 | -32.2711 | 5.00E-05 | 0.00014239 |
| MX1 | -6.96118 | -17.9161 | 5.00E-05 | 0.00014239 |
| HERC6 | -6.66821 | -19.3611 | 5.00E-05 | 0.00014239 |
| IFI6 | -6.5539 | -33.3361 | 5.00E-05 | 0.00014239 |
| IFI44L | -6.35925 | -7.84627 | 5.00E-05 | 0.00014239 |
| RSAD2 | -6.34871 | -9.1409 | 5.00E-05 | 0.00014239 |
| IFI44L | -6.18174 | -8.35974 | 5.00E-05 | 0.00014239 |
| XAF1 | -6.09109 | -21.3948 | 5.00E-05 | 0.00014239 |
| PARP12 | -6.05446 | -19.7542 | 5.00E-05 | 0.00014239 |
| IFI44L | -5.96757 | -8.08851 | 5.00E-05 | 0.00014239 |
| UBE2L6 | -5.95766 | -18.1069 | 5.00E-05 | 0.00014239 |
| fibronectin | -5.94132 | -4.65614 | 0.0005 | 0.00696984 |
| LAMP3 | -5.76223 | -3.75541 | 0.00715 | 0.0695686 |
| OAS3 | -5.58996 | -31.241 | 5.00E-05 | 0.00014239 |
| LGALS9 | -5.5438 | -19.6268 | 5.00E-05 | 0.00014239 |
| TRIM22 | -5.44544 | -20.4516 | 5.00E-05 | 0.00014239 |
| IFI6 | -5.4306 | -30.8592 | 5.00E-05 | 0.00014239 |
| HERC6 | -5.42298 | -24.9465 | 5.00E-05 | 0.00014239 |
| ETV7 | -5.39628 | -2.58129 | 0.0002 | 0.00264676 |
| IFI6 | -5.33734 | -30.3417 | 5.00E-05 | 0.00014239 |
| EPSTI1 | -5.2783 | -18.5231 | 5.00E-05 | 0.00014239 |
| IFI6 | -5.19506 | -29.76 | 5.00E-05 | 0.00014239 |
| PHF11 | -5.16508 | -4.64828 | 0.0019 | 0.0234083 |
| HSH2D | -5.15039 | -9.5073 | 5.00E-05 | 0.00014239 |
| RPS17 | -4.99601 | -6.54584 | 0.0009 | 0.0120786 |
| OAS2 | -4.98372 | -28.4385 | 5.00E-05 | 0.00014239 |
| CMPK2 | -4.97637 | -11.5977 | 5.00E-05 | 0.00014239 |
| IL12RB1 | -4.96782 | -0.116508 | 5.00E-05 | 0.00014239 |
| TRIM22 | -4.94736 | -21.6573 | 5.00E-05 | 0.00014239 |
| vimentin | -4.92624 | -17.8481 | 5.00E-05 | 0.00014239 |
| RSAD2 | -4.82277 | -14.4026 | 5.00E-05 | 0.00014239 |
| SLC15A3 | -4.78001 | -5.60362 | 5.00E-05 | 0.00014239 |
| XAF1 | -4.77155 | -1.71408 | 0.0002 | 0.00264676 |
| USP18 | -4.76923 | -18.2876 | 5.00E-05 | 0.00014239 |
| HSH2D | -4.65071 | -11.22 | 5.00E-05 | 0.00014239 |
| UBE2L6 | -4.55511 | -14.3524 | 5.00E-05 | 0.00014239 |
| ZBP1 | -4.44105 | -1.70444 | 5.00E-05 | 0.00014239 |
| TRIM22 | -4.42719 | -19.7122 | 5.00E-05 | 0.00014239 |
| RTP4 | -4.38384 | -8.77389 | 5.00E-05 | 0.00014239 |
| SAMD9L | -4.26014 | -22.8826 | 5.00E-05 | 0.00014239 |
| BATF2 | -4.21152 | -14.949 | 5.00E-05 | 0.00014239 |
| GJB1 | -4.08252 | -8.16642 | 5.00E-05 | 0.00014239 |
| OAS3 | -4.03812 | -24.5072 | 5.00E-05 | 0.00014239 |
| USP18 | -4.01118 | -19.3127 | 5.00E-05 | 0.00014239 |
| FAM213B | -3.83626 | -0.26097 | 0.0005 | 0.00696984 |
| XAF1 | -3.83118 | -22.2922 | 5.00E-05 | 0.00014239 |
| IFITM3 | -3.79441 | -24.2406 | 5.00E-05 | 0.00014239 |
| NLRC5 | -3.77045 | -18.6898 | 5.00E-05 | 0.00014239 |
| IFI16 | -3.75114 | -18.0952 | 5.00E-05 | 0.00014239 |
| OAS2 | -3.74423 | -23.2698 | 5.00E-05 | 0.00014239 |
| BTC | -3.64986 | -0.72752 | 0.0066 | 0.0652469 |
| BTC | -3.63846 | -0.80062 | 0.0067 | 0.0660542 |
| IFIT5 | -3.62635 | -21.1452 | 5.00E-05 | 0.00014239 |
| IFIH1 | -3.61464 | -21.4055 | 5.00E-05 | 0.00014239 |
| PARP9 | -3.60282 | -13.8622 | 5.00E-05 | 0.00014239 |
| IFI16 | -3.56982 | -18.6405 | 5.00E-05 | 0.00014239 |
| ZBP1 | -3.54638 | -1.29048 | 0.0001 | 0.00103288 |
| GJB1 | -3.51109 | -7.19006 | 5.00E-05 | 0.00014239 |
| GJB1 | -3.50415 | -9.1523 | 5.00E-05 | 0.00014239 |
| HERC6 | -3.44439 | -11.0106 | 5.00E-05 | 0.00014239 |
| OAS2 | -3.43494 | -21.9475 | 5.00E-05 | 0.00014239 |
| ZBP1 | -3.40346 | -3.04036 | 5.00E-05 | 0.00014239 |
| GTF2H2C | -3.38264 | -0.74479 | 0.0014 | 0.017914 |
| PARP12 | -3.37612 | -20.1182 | 5.00E-05 | 0.00014239 |
| USP18 | -3.34178 | -16.6636 | 5.00E-05 | 0.00014239 |
| RTP4 | -3.32597 | -6.92241 | 5.00E-05 | 0.00014239 |
| GJB1 | -3.308 | -6.83388 | 5.00E-05 | 0.00014239 |
| PDXDC1 | -3.29538 | -1.32189 | 5.00E-05 | 0.00014239 |
| IFIT5 | -3.28942 | -20.0255 | 5.00E-05 | 0.00014239 |
| CMPK2 | -3.2875 | -5.03388 | 5.00E-05 | 0.00014239 |
| GJB1 | -3.24547 | -9.5105 | 5.00E-05 | 0.00014239 |
| TNFSF13B | -3.2085 | -3.66093 | 0.00055 | 0.00764491 |
| OAS2 | -3.19397 | -20.9387 | 5.00E-05 | 0.00014239 |
| NLRC5 | -3.18705 | -16.338 | 5.00E-05 | 0.00014239 |
| PARN | -3.18448 | -0.4523 | 0.0006 | 0.0082879 |
| IL12RB1 | -3.15814 | 0.178751 | 0.0001 | 0.00103288 |
| ETV7 | -3.12616 | -1.39006 | 0.0002 | 0.00264676 |
| CMPK2 | -3.11601 | -15.8149 | 5.00E-05 | 0.00014239 |
| SAMHD1 | -3.09291 | -15.4532 | 5.00E-05 | 0.00014239 |
| PARP12 | -3.02879 | -11.0444 | 5.00E-05 | 0.00014239 |
| SP110 | -3.02021 | -19.5179 | 5.00E-05 | 0.00014239 |
| IFITM1 | -2.99695 | -20.6593 | 5.00E-05 | 0.00014239 |
| HELZ2 | -2.95046 | -20.3452 | 5.00E-05 | 0.00014239 |
| GJB1 | -2.93272 | -7.93462 | 5.00E-05 | 0.00014239 |
| DDX58 | -2.84212 | -19.7208 | 5.00E-05 | 0.00014239 |
| USP18 | -2.83837 | -17.7078 | 5.00E-05 | 0.00014239 |
| EPSTI1 | -2.81549 | -11.0028 | 5.00E-05 | 0.00014239 |
| HSH2D | -2.80102 | -7.12443 | 5.00E-05 | 0.00014239 |
| HERC5 | -2.76347 | -18.1991 | 5.00E-05 | 0.00014239 |
| LAMP3 | -2.74727 | -8.36861 | 5.00E-05 | 0.00014239 |
| LAMP3 | -3.99267 | -9.24361 | 5.00E-05 | 0.00101889 |
| LGALS9 | -3.97597 | -18.9491 | 5.00E-05 | 0.00101889 |
| GJB1 | -3.97503 | -8.36158 | 5.00E-05 | 0.00101889 |
| BTC | -3.97208 | -2.42295 | 0.00275 | 0.0328687 |
| EXOC3L1 | -3.96798 | -6.79517 | 5.00E-05 | 0.00101889 |
| SAMHD1 | -3.94592 | -11.5118 | 5.00E-05 | 0.00101889 |
| PDXDC1 | -3.94385 | -1.89752 | 5.00E-05 | 0.00101889 |
| PHF11 | -3.94196 | -3.33012 | 0.0086 | 0.0814484 |
| TNFSF10 | -3.9409 | -15.3398 | 5.00E-05 | 0.00101889 |
| IFI16 | -3.92968 | -17.8332 | 5.00E-05 | 0.00101889 |
| GJB1 | -3.91944 | -9.05021 | 5.00E-05 | 0.00101889 |
| IFI16 | -3.90444 | -17.6792 | 5.00E-05 | 0.00101889 |
| DDX60 | -3.0304 | -15.5569 | 5.00E-05 | 0.00101889 |
| PARP12 | -3.02568 | -15.0962 | 5.00E-05 | 0.00101889 |
| PARP12 | -3.02538 | -8.3596 | 5.00E-05 | 0.00101889 |
| GBP4 | -3.02433 | -9.7635 | 5.00E-05 | 0.00101889 |
| MLXIPL | -3.00703 | -8.41536 | 5.00E-05 | 0.00101889 |
| ONECUT3 | -3.00663 | -5.36594 | 5.00E-05 | 0.00101889 |
| IFI44 | -3.00479 | -14.6127 | 5.00E-05 | 0.00101889 |
| ONECUT3 | -3.0038 | -5.21929 | 5.00E-05 | 0.00101889 |
| CYP2J2 | -3.00108 | -3.96779 | 0.00085 | 0.0123456 |
| MUC5B | -2.99871 | -13.4653 | 5.00E-05 | 0.00101889 |
| IFI44 | -2.99844 | -14.4948 | 5.00E-05 | 0.00101889 |
| RTP4 | -2.99546 | -10.8429 | 5.00E-05 | 0.00101889 |
| CLDN2 | -2.99038 | -12.9936 | 5.00E-05 | 0.00101889 |
| IFITM1 | -2.98792 | -15.4562 | 5.00E-05 | 0.00101889 |
| HSD11B2 | -2.64875 | -6.49508 | 5.00E-05 | 0.00101889 |
| BATF2 | -2.64857 | -12.3839 | 5.00E-05 | 0.00101889 |
| H6PD | -2.64815 | -12.1981 | 5.00E-05 | 0.00101889 |
| CD74 | -2.6438 | -11.7067 | 5.00E-05 | 0.00101889 |
| SAMD9 | -2.64358 | -13.6082 | 5.00E-05 | 0.00101889 |
| PLSCR1 | -2.64312 | -13.0804 | 5.00E-05 | 0.00101889 |
| EIF2AK2 | -2.64206 | -11.2453 | 5.00E-05 | 0.00101889 |
| SLC15A3 | -2.63947 | -9.0195 | 5.00E-05 | 0.00101889 |
| PLEKHA4 | -2.6379 | -11.8424 | 5.00E-05 | 0.00101889 |
| PARP14 | -2.63487 | -13.5216 | 5.00E-05 | 0.00101889 |
| ACOT1 | -2.41708 | -2.85454 | 0.0011 | 0.0153257 |
| RNF144A | -2.41403 | -10.2301 | 5.00E-05 | 0.00101889 |
| PRSS8 | -2.41192 | -4.32482 | 5.00E-05 | 0.00101889 |
| IGFL2 | -2.41141 | -8.11287 | 5.00E-05 | 0.00101889 |
| CYP2J2 | -2.40946 | -3.01324 | 0.00415 | 0.0458077 |
| GBP1 | -2.40769 | -10.5942 | 5.00E-05 | 0.00101889 |
| KLK7 | -2.4075 | -6.64092 | 5.00E-05 | 0.00101889 |
| SP100 | -2.40552 | -11.6739 | 5.00E-05 | 0.00101889 |
| IFIT3 | -2.4008 | -12.4708 | 5.00E-05 | 0.00101889 |
| EGR2 | -2.40071 | -5.54997 | 5.00E-05 | 0.00101889 |
| ENTPD2 | -2.39988 | -7.62673 | 5.00E-05 | 0.00101889 |
| IGFL2 | -2.39905 | -8.23127 | 5.00E-05 | 0.00101889 |
| PLSCR1 | -2.39814 | -11.8438 | 5.00E-05 | 0.00101889 |
| ASS1 | -2.39356 | -11.4495 | 5.00E-05 | 0.00101889 |
| MLXIPL | -2.39244 | -7.88548 | 5.00E-05 | 0.00101889 |
| BATF2 | -2.39218 | -6.67351 | 5.00E-05 | 0.00101889 |
| C1orf210 | -2.39022 | -3.49948 | 5.00E-05 | 0.00101889 |
| SCIN | -2.02886 | -3.39651 | 0.00015 | 0.00273852 |
| KLK5 | -2.0279 | -2.41363 | 0.00105 | 0.0147495 |
| MGAT3 | -2.02592 | -3.35452 | 5.00E-05 | 0.00101889 |
| DDIT4 | -2.02562 | -9.8451 | 5.00E-05 | 0.00101889 |
| C2orf54 | -2.02488 | -2.14336 | 0.00285 | 0.0338567 |
| CXCR3 | -2.0246 | -1.96755 | 0.00165 | 0.0215983 |
| SCIN | -2.02424 | -3.39944 | 0.00015 | 0.00273852 |
| SULT2B1 | -2.02419 | -7.37501 | 5.00E-05 | 0.00101889 |
| PRSS8 | -2.02406 | -3.54666 | 0.00045 | 0.0071621 |
| TRIM5 | -2.02267 | -9.13312 | 5.00E-05 | 0.00101889 |
| PRSS8 | -2.02221 | -4.09565 | 5.00E-05 | 0.00101889 |
| TMPRSS3 | -2.02155 | -3.54732 | 5.00E-05 | 0.00101889 |
| ACOT1 | -2.02057 | -2.28505 | 0.00895 | 0.084097 |
| UNC5B | -2.01864 | -9.18632 | 5.00E-05 | 0.00101889 |
| TTBK1 | -2.01846 | -7.92826 | 5.00E-05 | 0.00101889 |
| DDX60L | -2.01804 | -10.1453 | 5.00E-05 | 0.00101889 |
| HSH2D | -2.01707 | -3.95896 | 5.00E-05 | 0.00101889 |
| PARP10 | -2.01513 | -10.4739 | 5.00E-05 | 0.00101889 |
| LMF1 | -2.0136 | -5.76844 | 5.00E-05 | 0.00101889 |
| ARL6IP6 | -2.01218 | -2.60197 | 0.003 | 0.0353117 |
| ARL6IP6 | -2.01111 | -2.60251 | 0.0043 | 0.0470996 |

RNA-seq results showed a list of up-regulated genes (215) in BxPC-3 cells overexpression LECT2

| Symbol | logFC | test_stat | p_value | adj.P.Val |
| --- | --- | --- | --- | --- |
| E-cadherin | 8.18035 | 30.6676 | 5.00E-05 | 0.00101889 |
| MX1 | 8.09731 | 19.5514 | 5.00E-05 | 0.00101889 |
| UBE2L6 | 8.03007 | 15.8357 | 5.00E-05 | 0.00101889 |
| XAF1 | 7.9917 | 17.9546 | 5.00E-05 | 0.00101889 |
| IFI6 | 7.65701 | 33.6744 | 5.00E-05 | 0.00101889 |
| LAMP3 | 7.3946 | 4.51364 | 0.0005 | 0.00784634 |
| IFI44L | 7.39048 | 8.47169 | 5.00E-05 | 0.00101889 |
| IFI6 | 7.35735 | 33.4615 | 5.00E-05 | 0.00101889 |
| UBE2L6 | 7.22815 | 14.2221 | 5.00E-05 | 0.00101889 |
| IFI6 | 7.21507 | 32.9062 | 5.00E-05 | 0.00101889 |
| UBE2L6 | 7.05191 | 18.5833 | 5.00E-05 | 0.00101889 |
| C1S | 6.9357 | 5.7179 | 0.00385 | 0.0431411 |
| SLC15A3 | 6.90599 | 7.61996 | 5.00E-05 | 0.00101889 |
| MX1 | 6.84147 | 25.8615 | 5.00E-05 | 0.00101889 |
| XAF1 | 6.82685 | 15.3327 | 5.00E-05 | 0.00101889 |
| UBE2L6 | 6.77867 | 13.3393 | 5.00E-05 | 0.00101889 |
| SLC15A3 | 6.71968 | 7.36819 | 5.00E-05 | 0.00101889 |
| IFI6 | 6.53372 | 31.1528 | 5.00E-05 | 0.00101889 |
| IL12RB1 | 6.36306 | 1.01569 | 5.00E-05 | 0.00101889 |
| UBE2L6 | 6.24999 | 16.4038 | 5.00E-05 | 0.00101889 |
| CMPK2 | 6.22853 | 19.8342 | 5.00E-05 | 0.00101889 |
| OAS2 | 6.17927 | 29.0449 | 5.00E-05 | 0.00101889 |
| TRIM22 | 6.17067 | 19.6302 | 5.00E-05 | 0.00101889 |
| PARP12 | 6.1649 | 17.3819 | 5.00E-05 | 0.00101889 |
| LGALS9 | 6.06928 | 22.4322 | 5.00E-05 | 0.00101889 |
| OAS2 | 5.98816 | 28.3809 | 5.00E-05 | 0.00101889 |
| PHF11 | 5.95415 | 5.11492 | 0.00215 | 0.0269164 |
| OAS2 | 5.93831 | 28.1112 | 5.00E-05 | 0.00101889 |
| LGALS9 | 5.89013 | 17.7446 | 5.00E-05 | 0.00101889 |
| IFI16 | 5.88618 | 18.4372 | 5.00E-05 | 0.00101889 |
| IFI16 | 5.86094 | 18.3386 | 5.00E-05 | 0.00101889 |
| SLC15A3 | 5.83909 | 6.24113 | 5.00E-05 | 0.00101889 |
| C1R | 5.82635 | 9.20432 | 5.00E-05 | 0.00101889 |
| CD74 | 5.81289 | 19.4326 | 5.00E-05 | 0.00101889 |
| C1R | 5.72659 | 8.41669 | 5.00E-05 | 0.00101889 |
| RPS17 | 5.63241 | 8.26605 | 5.00E-05 | 0.00101889 |
| IL4I1 | 5.46591 | 2.82958 | 0.00995 | 0.091316 |
| RPS17 | 5.46384 | 8.32696 | 5.00E-05 | 0.00101889 |
| RPS17 | 5.43369 | 6.37146 | 0.00125 | 0.0170878 |
| C3 | 5.41179 | 17.9513 | 5.00E-05 | 0.00101889 |
| USP18 | 5.34523 | 16.9777 | 5.00E-05 | 0.00101889 |
| ETV7 | 5.29963 | 2.75282 | 5.00E-05 | 0.00101889 |
| BATF2 | 5.29532 | 16.7097 | 5.00E-05 | 0.00101889 |
| FAM213B | 5.28538 | 1.00903 | 0.0016 | 0.0210662 |
| EPSTI1 | 5.27684 | 15.5793 | 5.00E-05 | 0.00101889 |
| C3 | 5.26697 | 17.4559 | 5.00E-05 | 0.00101889 |
| C1S | 5.26582 | 4.3287 | 0.00385 | 0.0431411 |
| SAA2 | 5.20357 | 3.10743 | 0.0046 | 0.0497377 |
| OAS3 | 5.19833 | 25.9747 | 5.00E-05 | 0.00101889 |
| LGALS9 | 5.17021 | 19.0414 | 5.00E-05 | 0.00101889 |
| C1S | 5.12925 | 9.07951 | 5.00E-05 | 0.00101889 |
| SAA2 | 5.12513 | 3.05912 | 0.0046 | 0.0497377 |
| SAMD9L | 5.11893 | 22.1649 | 5.00E-05 | 0.00101889 |
| SAMD9L | 5.03499 | 22.9211 | 5.00E-05 | 0.00101889 |
| IFI16 | 5.02178 | 19.0959 | 5.00E-05 | 0.00101889 |
| HERC6 | 5.01789 | 12.7241 | 5.00E-05 | 0.00101889 |
| C3 | 5.00381 | 16.552 | 5.00E-05 | 0.00101889 |
| RSAD2 | 4.99098 | 6.76427 | 5.00E-05 | 0.00101889 |
| BATF2 | 4.98751 | 14.4364 | 5.00E-05 | 0.00101889 |
| IL12RB1 | 4.95959 | 1.58971 | 5.00E-05 | 0.00101889 |
| USP18 | 4.93665 | 20.5599 | 5.00E-05 | 0.00101889 |
| RSAD2 | 4.9132 | 13.9193 | 5.00E-05 | 0.00101889 |
| ZC3H12C | 4.89913 | 4.94863 | 5.00E-05 | 0.00101889 |
| C1S | 4.86848 | 4.00034 | 0.00385 | 0.0431411 |
| IFI16 | 4.84046 | 19.6529 | 5.00E-05 | 0.00101889 |
| C3 | 4.83394 | 18.5014 | 5.00E-05 | 0.00101889 |
| SAA2 | 4.82833 | 2.8809 | 0.0047 | 0.0505423 |
| BATF2 | 4.82591 | 15.1955 | 5.00E-05 | 0.00101889 |
| RPS17 | 4.82469 | 6.93357 | 5.00E-05 | 0.00101889 |
| IL12RB1 | 4.80974 | 1.54002 | 5.00E-05 | 0.00101889 |
| SAA1 | 4.7825 | 4.23811 | 0.00015 | 0.00273852 |
| OAS2 | 4.74866 | 23.1525 | 5.00E-05 | 0.00101889 |
| PARP9 | 4.71879 | 14.4642 | 5.00E-05 | 0.00101889 |
| PHF11 | 4.70596 | 7.47014 | 5.00E-05 | 0.00101889 |
| APOBEC3G | 4.68989 | 4.1615 | 0.00015 | 0.00273852 |
| C3 | 4.68912 | 17.9265 | 5.00E-05 | 0.00101889 |
| SLC15A3 | 4.67881 | 4.94678 | 5.00E-05 | 0.00101889 |
| CXCL5 | 4.65725 | 23.327 | 5.00E-05 | 0.00101889 |
| RPS17 | 4.65612 | 6.93741 | 5.00E-05 | 0.00101889 |
| EPSTI1 | 4.65148 | 16.6716 | 5.00E-05 | 0.00101889 |
| α-catenin | 4.64838 | 21.2417 | 5.00E-05 | 0.00101889 |
| SAA1 | 4.63899 | 4.10648 | 0.00015 | 0.00273852 |
| ZC3H12C | 4.59111 | 4.63922 | 5.00E-05 | 0.00101889 |
| FAM213B | 4.54744 | 1.43273 | 0.00035 | 0.00577121 |
| HSH2D | 4.54609 | 7.20857 | 5.00E-05 | 0.00101889 |
| SAMHD1 | 4.32625 | 14.1841 | 5.00E-05 | 0.00101889 |
| IFIH1 | 4.32468 | 20.3978 | 5.00E-05 | 0.00101889 |
| SAA2 | 4.28502 | 4.51104 | 0.00085 | 0.0123456 |
| PARP12 | 4.27448 | 20.72 | 5.00E-05 | 0.00101889 |
| USP18 | 4.26725 | 17.6793 | 5.00E-05 | 0.00101889 |
| SLC15A3 | 4.26652 | 4.62835 | 5.00E-05 | 0.00101889 |
| POU2F2 | 4.25842 | 8.33901 | 5.00E-05 | 0.00101889 |
| SLCO5A1 | 4.2583 | 17.825 | 5.00E-05 | 0.00101889 |
| PHF11 | 4.24963 | 6.67872 | 5.00E-05 | 0.00101889 |
| SIRT4 | 4.24037 | 2.39102 | 0.0001 | 0.00190938 |
| CXCL5 | 4.23967 | 21.4587 | 5.00E-05 | 0.00101889 |
| GBP1 | 4.23457 | 19.1787 | 5.00E-05 | 0.00101889 |
| PARP9 | 4.21917 | 12.5914 | 5.00E-05 | 0.00101889 |
| C1R | 4.20151 | 6.57784 | 5.00E-05 | 0.00101889 |
| HIST1H1B | 4.18726 | 4.50503 | 0.0099 | 0.0909806 |
| SP110 | 4.17926 | 19.9982 | 5.00E-05 | 0.00101889 |
| BST2 | 4.16995 | 20.4542 | 5.00E-05 | 0.00101889 |
| APOBEC3G | 4.14986 | 3.67234 | 0.00015 | 0.00273852 |
| C3 | 4.13181 | 16.7614 | 5.00E-05 | 0.00101889 |
| CXCL5 | 4.10179 | 20.8328 | 5.00E-05 | 0.00101889 |
| C1R | 4.10175 | 5.98163 | 5.00E-05 | 0.00101889 |
| CXCL5 | 4.08507 | 20.5439 | 5.00E-05 | 0.00101889 |
| DUOXA2 | 4.07684 | 7.78627 | 5.00E-05 | 0.00101889 |
| DUOXA2 | 4.07672 | 7.78767 | 5.00E-05 | 0.00101889 |
| TNFSF13B | 4.0618 | 4.93202 | 0.00015 | 0.00273852 |
| IFITM1 | 4.04726 | 20.6457 | 5.00E-05 | 0.00101889 |
| CD74 | 4.04633 | 18.2323 | 5.00E-05 | 0.00101889 |
| POU2F2 | 4.04568 | 7.78672 | 5.00E-05 | 0.00101889 |
| C1S | 4.0412 | 9.26862 | 5.00E-05 | 0.00101889 |
| HERC6 | 4.04096 | 19.479 | 5.00E-05 | 0.00101889 |
| TRANK1 | 3.86448 | 19.031 | 5.00E-05 | 0.00101889 |
| IFITM3 | 3.83788 | 19.1194 | 5.00E-05 | 0.00101889 |
| GBP1 | 3.83357 | 16.9502 | 5.00E-05 | 0.00101889 |
| DUOXA2 | 3.82849 | 6.47014 | 5.00E-05 | 0.00101889 |
| HIST1H1B | 3.82236 | 3.91964 | 0.00205 | 0.0258672 |
| SLC15A3 | 3.79822 | 3.92134 | 0.0005 | 0.00784634 |
| APOBEC3G | 3.79107 | 3.33289 | 0.00025 | 0.00428971 |
| IFIT5 | 3.78414 | 16.9881 | 5.00E-05 | 0.00101889 |
| CCL20 | 3.78386 | 17.0536 | 5.00E-05 | 0.00101889 |
| IFIT1 | 3.78172 | 19.3956 | 5.00E-05 | 0.00101889 |
| NOMO1 | 3.78052 | 6.73586 | 5.00E-05 | 0.00101889 |
| C9orf129 | 3.7636 | 2.79208 | 0.0019 | 0.0242848 |
| XAF1 | 3.75704 | 1.60767 | 0.00045 | 0.0071621 |
| ZBP1 | 3.75418 | 3.12165 | 0.00025 | 0.00428971 |
| NAPRT | 3.75227 | 5.64439 | 5.00E-05 | 0.00101889 |
| POU2F2 | 3.75046 | 8.51021 | 5.00E-05 | 0.00101889 |
| IFIT1 | 3.73747 | 19.3093 | 5.00E-05 | 0.00101889 |
| C1R | 3.7349 | 5.85096 | 5.00E-05 | 0.00101889 |
| IFIT1 | 3.73413 | 19.0889 | 5.00E-05 | 0.00101889 |
| IFITM1 | 3.72779 | 18.9862 | 5.00E-05 | 0.00101889 |
| DUOX2 | 3.7205 | 14.5034 | 5.00E-05 | 0.00101889 |
| CCL20 | 3.71864 | 16.7722 | 5.00E-05 | 0.00101889 |
| APOL3 | 3.71227 | 7.72942 | 5.00E-05 | 0.00101889 |
| APOBEC3G | 2.40982 | 2.03333 | 0.00245 | 0.0298908 |
| CSF1 | 2.40665 | 10.7224 | 5.00E-05 | 0.00101889 |
| GMFG | 2.40664 | 3.62444 | 0.00015 | 0.00273852 |
| SH2D1B | 2.40218 | 5.06227 | 5.00E-05 | 0.00101889 |
| TRIM21 | 2.39879 | 12.1468 | 5.00E-05 | 0.00101889 |
| CTSS | 2.39859 | 11.9113 | 5.00E-05 | 0.00101889 |
| RTP4 | 2.38852 | 9.78817 | 5.00E-05 | 0.00101889 |
| DTX3L | 2.38822 | 9.00785 | 5.00E-05 | 0.00101889 |
| ISG15 | 2.38779 | 12.3957 | 5.00E-05 | 0.00101889 |
| BIRC3 | 2.38707 | 12.2791 | 5.00E-05 | 0.00101889 |
| SLC2A5 | 2.38641 | 5.91639 | 5.00E-05 | 0.00101889 |
| SECTM1 | 2.38301 | 11.3775 | 5.00E-05 | 0.00101889 |
| RPS18 | 2.38039 | 5.45432 | 5.00E-05 | 0.00101889 |
| FLT3LG | 2.38026 | 3.27497 | 5.00E-05 | 0.00101889 |
| IRF9 | 2.37893 | 11.8686 | 5.00E-05 | 0.00101889 |
| UBA7 | 2.37162 | 10.494 | 5.00E-05 | 0.00101889 |
| C1S | 2.37132 | 5.32068 | 5.00E-05 | 0.00101889 |
| CYP2J2 | 2.36488 | 3.03478 | 0.00325 | 0.0376343 |
| PLEKHA4 | 2.36279 | 10.614 | 5.00E-05 | 0.00101889 |
| CXCR4 | 2.36206 | 2.56783 | 0.00535 | 0.0560272 |
| SH2D1B | 2.36027 | 4.63833 | 5.00E-05 | 0.00101889 |
| SP100 | 2.35933 | 11.4699 | 5.00E-05 | 0.00101889 |
| CTSS | 2.35722 | 11.2406 | 5.00E-05 | 0.00101889 |
| CX3CL1 | 2.35434 | 5.26864 | 5.00E-05 | 0.00101889 |
| FBXO6 | 2.10512 | 5.95465 | 5.00E-05 | 0.00101889 |
| PHOSPHO2-KLHL23 | 2.10076 | 2.74743 | 0.00155 | 0.0205217 |
| ABR | 2.09982 | 3.25071 | 5.00E-05 | 0.00101889 |
| GMFG | 2.09922 | 3.41954 | 0.00015 | 0.00273852 |
| CYTIP | 2.09711 | 3.18003 | 0.00095 | 0.0135715 |
| GIMAP2 | 2.09705 | 4.0392 | 5.00E-05 | 0.00101889 |
| CCL20 | 2.09492 | 10.2906 | 5.00E-05 | 0.00101889 |
| PARP9 | 2.09473 | 5.98222 | 5.00E-05 | 0.00101889 |
| LGALS9 | 2.09331 | 7.40015 | 5.00E-05 | 0.00101889 |
| C1R | 2.09145 | 9.02685 | 5.00E-05 | 0.00101889 |
| TDRD7 | 2.08543 | 10.1048 | 5.00E-05 | 0.00101889 |
| CSF1 | 2.08048 | 9.2068 | 5.00E-05 | 0.00101889 |
| GIMAP2 | 2.07619 | 3.7191 | 5.00E-05 | 0.00101889 |
| SIRT4 | 2.07561 | 3.42473 | 5.00E-05 | 0.00101889 |
| SP100 | 2.07208 | 10.0984 | 5.00E-05 | 0.00101889 |
| RGCC | 2.0692 | 8.6919 | 5.00E-05 | 0.00101889 |
| HIST1H4H | 2.06872 | 2.60528 | 0.0074 | 0.0723727 |
| APOL1 | 2.06807 | 10.5316 | 5.00E-05 | 0.00101889 |
| C1S | 2.06722 | 8.64602 | 5.00E-05 | 0.00101889 |
| ICAM1 | 2.06333 | 8.30842 | 5.00E-05 | 0.00101889 |
| ANKRD1 | 2.06302 | 6.46475 | 5.00E-05 | 0.00101889 |
| NEURL3 | 2.06101 | 6.41769 | 5.00E-05 | 0.00101889 |
| GBP2 | 2.06029 | 10.2779 | 5.00E-05 | 0.00101889 |
| C4BPA | 2.05973 | 3.45315 | 0.00025 | 0.00428971 |
| C4BPA | 2.05824 | 3.6855 | 5.00E-05 | 0.00101889 |
| MB | 2.05758 | 3.10173 | 0.00015 | 0.00273852 |
| ODF3B | 2.05294 | 8.16563 | 5.00E-05 | 0.00101889 |
| TMEM156 | 2.05037 | 1.6155 | 0.00465 | 0.0501365 |
| IRF1 | 2.04993 | 10.164 | 5.00E-05 | 0.00101889 |
| KISS1 | 2.03965 | 5.71403 | 5.00E-05 | 0.00101889 |
| PTPRM | 2.03955 | 3.00157 | 5.00E-05 | 0.00101889 |
| IL32 | 2.03638 | 9.92293 | 5.00E-05 | 0.00101889 |
| HDAC9 | 2.03624 | 7.69052 | 5.00E-05 | 0.00101889 |
| WARS | 2.03601 | 10.6278 | 5.00E-05 | 0.00101889 |
| PHOSPHO2-KLHL23 | 2.03565 | 2.67409 | 0.00205 | 0.0258672 |
| ADORA2A | 2.03218 | 3.79452 | 5.00E-05 | 0.00101889 |
| CXCL8 | 2.03177 | 9.24159 | 5.00E-05 | 0.00101889 |
| HSH2D | 2.02935 | 6.62256 | 5.00E-05 | 0.00101889 |
| TRIM5 | 2.0292 | 9.18945 | 5.00E-05 | 0.00101889 |
| GSDMB | 2.02915 | 8.10283 | 5.00E-05 | 0.00101889 |
| VSNL1 | 2.02716 | 3.82107 | 5.00E-05 | 0.00101889 |
| ABR | 2.02691 | 3.04952 | 5.00E-05 | 0.00101889 |
| CX3CL1 | 2.02112 | 4.48705 | 5.00E-05 | 0.00101889 |
| TRIM14 | 2.0194 | 8.70651 | 5.00E-05 | 0.00101889 |
| C6orf222 | 2.01628 | 5.21848 | 5.00E-05 | 0.00101889 |
| BIRC3 | 2.01598 | 10.3541 | 5.00E-05 | 0.00101889 |
| PHF11 | 2.01219 | 5.63685 | 5.00E-05 | 0.00101889 |
| WARS | 2.01133 | 10.2536 | 5.00E-05 | 0.00101889 |
| FLT3LG | 2.0052 | 2.8007 | 5.00E-05 | 0.00101889 |
| PTPRM | 2.00498 | 2.93858 | 5.00E-05 | 0.00101889 |
| FZD2 | 2.00308 | 4.54723 | 5.00E-05 | 0.00101889 |
| ANKRD1 | 2.00292 | 6.35361 | 5.00E-05 | 0.00101889 |
| CIITA | 2.00213 | 3.53756 | 5.00E-05 | 0.00101889 |
